# Syndecan-4 affects myogenesis via Rac1-mediated actin remodeling and exhibits copy-number amplification and increased expression in human rhabdomyosarcoma tumors

**DOI:** 10.1007/s00018-021-04121-0

**Published:** 2022-02-07

**Authors:** Kitti Szabo, Daniel Varga, Attila Gergely Vegh, Ning Liu, Xue Xiao, Lin Xu, Laszlo Dux, Miklos Erdelyi, Laszlo Rovo, Aniko Keller-Pinter

**Affiliations:** 1 Department of Biochemistry, Albert Szent-Gyorgyi Medical School, University of Szeged, Szeged, Hungary; 2 Department of Optics and Quantum Electronics, Faculty of Science and Informatics, University of Szeged, Szeged, Hungary; 3 Institute of Biophysics, Biological Research Centre, Eotvos Lorand Research Network (ELKH), Szeged, Hungary; 4 Department of Molecular Biology, University of Texas Southwestern Medical Center, Dallas, TX USA; 5 Quantitative Biomedical Research Center, Department of Population and Data Sciences, University of Texas Southwestern Medical Center, Dallas, TX USA; 6 Department of Oto-Rhino-Laryngology and Head- Neck Surgery, University of Szeged, Szeged, Hungary

**Keywords:** Syndecan-4, Proteoglycan, Actin, Rac1, Muscle differentiation, Myoblast fusion, Rhabdomyosarcoma, dSTORM superresolution microscopy, Atomic force microscopy

## Abstract

Skeletal muscle demonstrates a high degree of regenerative capacity repeating the embryonic myogenic program under strict control. Rhabdomyosarcoma is the most common sarcoma in childhood and is characterized by impaired muscle differentiation. In this study, we observed that silencing the expression of syndecan-4, the ubiquitously expressed transmembrane heparan sulfate proteoglycan, significantly enhanced myoblast differentiation, and fusion. During muscle differentiation, the gradually decreasing expression of syndecan-4 allows the activation of Rac1, thereby mediating myoblast fusion. Single-molecule localized superresolution direct stochastic optical reconstruction microscopy (dSTORM) imaging revealed nanoscale changes in actin cytoskeletal architecture, and atomic force microscopy showed reduced elasticity of syndecan-4-knockdown cells during fusion. Syndecan-4 copy-number amplification was observed in 28% of human fusion-negative rhabdomyosarcoma tumors and was accompanied by increased syndecan-4 expression based on RNA sequencing data. Our study suggests that syndecan-4 can serve as a tumor driver gene in promoting rabdomyosarcoma tumor development. Our results contribute to the understanding of the role of syndecan-4 in skeletal muscle development, regeneration, and tumorigenesis.

**Supplementary Information:**

The online version contains supplementary material available at 10.1007/s00018-021-04121-0.

## Introduction

A population of resident stem cells (i.e., satellite cells) accounts for skeletal muscle plasticity, maintenance, and regeneration [1, 2]. Satellite cells are mitotically and physiologically quiescent in healthy muscles until stimulated by local damage. Accordingly, after a skeletal muscle injury, an intense regenerative program is initiated. The activated satellite cells migrate to the site of injury and become committed myoblasts, after which cell–cell fusion occurs, eventually creating multinucleated syncytium [3]. The transcription factors that regulate myogenesis and muscle differentiation include members of the MyoD family [MyoD, Myf5, MRF4, and MyoG (myogenin)], also known as myogenic regulatory factors (MRFs). MRFs appear in distinctive spatial and temporal patterns during embryonic development and regeneration of striated muscle. Myf5 and MyoD are expressed earlier, whereas MyoG and MRF4 are expressed later in somatic cells during limb development and differentiation of in vitro cell cultures [4].

At cellular level, the fusion of mononucleated myogenic cells is characterized by the alignment of myoblasts and/or myotube membranes followed by rearrangements of the actin cytoskeleton at the contact sites [5, 6]. The composition of the cell membrane also changes during fusion, and the phosphatidylserine content of the inner part of the lipid bilayer turns toward the outer part [7]. Eventually, myoblasts fuse by breaking down the cell membrane. As cell fusion can be observed in several processes, it can be encountered not only during myogenesis, but also during the formation of osteoclasts, syncytiotrophoblasts, and tumor cells.

The key intracellular components that act downstream of cell adhesion molecules to control the continuous and dynamic rearrangement of the actin cytoskeleton are the members of the Rho family of small GTPases, among which the best characterized members are RhoA, Rac1 (Ras-related C3 botulinum toxin substrate 1), and Cdc42 [8, 9]. Small GTPases function as molecular switches cycling between an active GTP-bound and an inactive GDP-bound conformation. The GTP-loaded forms interact with effector proteins, inducing downstream signaling events. Several studies indicate that Rac1 is a central regulator of myoblast fusion in *Drosophila* [10, 11]; furthermore, it has been reported that Rac1 and Cdc42 are essential for myoblast fusion in vertebrates [12]. The levels of Rac1-GTP increase at the site of fusion, and constitutively active Rac1 induces myoblast fusion [13]. In contrast, because active RhoA antagonizes Rac1-GTP, the expression of constitutively active RhoA reduces the fusion of myoblasts [14].

The actin cytoskeleton determines cell shape, cell motility, and intracellular transport, allowing the cell to flexibly adapt to external effects. In resting cells such as myotubes, actin filaments form a cortical actin network at the periphery of the cell [6]. During cell–cell fusion, protrusions of the membrane, sheet-like lamellipodia or finger-like filopodia, are formed. Data obtained from studies on *Drosophila* suggest that in the fusion-competent myoblasts, the development of an actin spike from actin filaments is also required [11]. However, in mammalian cells, finger-like actin-based protrusions are formed on fusion-competent myoblasts [15]. The Arp2/3 complex and the formins such as Dia are responsible for the nucleation of actin polymerization. The Arp2/3 complex, Rac1 and Cdc42, initiates the formation of a new filament by attaching it to the side of existing actin filaments at an angle of 70° to the original filament, and the Rho-effector Dia linearly extends the actin filaments [9]. Rho GTPases also play a role in regulating the activity of cofilin, which allows actin depolymerization. Phosphorylated cofilin (Ser3) is inactive, whereas the unphosphorylated form is active and catalyzes the depolymerization of actin filaments. The Rac1/p21-activated kinase (PAK)1–4 or RhoA/Rho kinase (ROCK)-induced activation of LIM kinase (LIMK)1, 2 regulates the phosphorylation of cofilin [16].

Syndecans are transmembrane proteoglycans that play multiple structural and signaling roles and are composed of a conserved variable N-terminal extracellular, transmembrane, and a variable C-terminal cytoplasmic domain [17]. In vertebrates, four types of syndecans are distinguished, whereas invertebrates exhibit only one type of syndecan [18]. With the exception of syndecan-4, which is expressed ubiquitously, their localization is rather tissue specific in vertebrates. Syndecan-1 can be observed on epithelial cells and leukocytes, primarily on plasma cells, syndecan-2 is characteristic for mesenchymal cells and developing neural tissue, and syndecan-3 can be detected alongside neural tissue in the developing skeletal muscle system [19]. Heparan sulfate chains are linked to the ectodomain of each syndecan, and additional chondroitin sulfate chains are present for syndecan-1 and syndecan-3 [19].

Syndecan-4 is a cell surface marker of quiescent and activated satellite cells [20]. The heparan sulfate chains of syndecan-4 interact with fibronectin [21] and bind different growth factors such as FGF2 [22] and promyostatin in myoblasts [23]. Syndecan-4 connects the extracellular matrix and cytoskeleton and participates in multiple biological processes such as cell–matrix adhesion [24], cytokinesis [25], cell migration and cell polarity [25–28], mechanotransduction [29], and endocytosis [30]. The cytoplasmic domain of syndecan-4 contains a PIP2 (phosphatidylinositol 4,5-bisphosphate)-binding site, and it also binds and activates protein kinase C alpha (PKCα) [24]. Syndecan-4 is connected to the actin cytoskeleton through α-actinin [31] and also regulates intracellular calcium level and distribution [27, 32]. Syndecan-4 regulates Rac1 activity, considering that the level of Rac1-GTP was increased in syndecan-4-KO mice [33]. Syndecan-4 binds T-cell lymphoma invasion and metastasis-inducing protein 1 (Tiam-1) in a phosphorylation-dependent manner, thereby regulating Rac1 activation and signaling [34]. Tiam1 is the primary guanine nucleotide exchange factor (GEF) activating Rac1 GTPase, and both the Ser179 residue and the EFYA motif (type II PDZ-binding motif) of syndecan-4 are involved in Tiam1 binding [34].

Studies reported that syndecan-4 KO mice exhibited a wound healing disorder and impaired angiogenesis [35], and impaired muscle regeneration [36]. At 5 days postinjury, syndecan-4 KO mice showed poorly organized, irregularly shaped and sized syncytia with variable, aberrant nucleation [36]. Syndecan-4 silenced cells do not or hardly bind FGF2, resulting in decreased FGF2-FGFR signaling and thus decreased cell proliferation, which allows muscle differentiation [37]. In the absence of syndecan-4, MyoD expression in satellite cells is reduced, and MyoD exhibits a highly cytoplasmic localization compared to that in the wild type, which exhibits nuclear localization. Moreover, the fiber-associated satellite cells of syndecan-4 KO mice did not form myotubes in vitro [36]. Earlier, we demonstrated that syndecan-4 silencing decreases mammalian myoblast proliferation by modulating myostatin signaling and G1/S transition [23], and also reduces myoblast migration [26, 27].

Rhabdomyosarcoma is the most common soft tissue sarcoma in childhood with skeletal muscle origin and is characterized by the impaired differentiation of muscle cells. Its incidence in young adults aged < 20 years has been reported to be 4.4/1 million per year in the USA [38]. From a molecular biological viewpoint, it distinguishes two major groups based on the PAX3/7-FOXO1 fusion gene status, namely, fusion-positive rhabdomyosarcoma (FPRMS), and fusion-negative rhabdomyosarcoma (FNRMS). Fusion-positive tumors contain either PAX3–FOXO1 fusion protein resulting from a chromosomal translocation t(2; 13)(q35; q14) or PAX7–FOXO1 fusion protein resulting from a translocation t(1; 13)(p36; q14) [39, 40]. In all other cases, the rhabdomyosarcomas are considered to be fusion negative.

Due to the unknown role of syndecan-4 in skeletal muscle-derived rhabdomyosarcomas, the rate of syndecan-4 copy-number amplification or gene loss in fusion-positive and fusion-negative rhabdomyosarcomas remains unclear. FNRMSs constitute a heterogeneous group, in which primarily point mutations have been identified; however, limited information on its pathogenesis exists. Syndecan-4 has been reported to be essential for skeletal muscle differentiation and syndecan-4 KO mice suffer from muscle regeneration defects [35]; however, the underlying mechanisms are still unclear. Moreover, the detailed changes of the actin cytoskeleton during mammalian myoblast fusion are not fully understood. In this study, we aimed to better understand the multiple roles of syndecan-4 in skeletal muscle. We demonstrate that silencing of syndecan-4 expression increases mammalian myoblast differentiation and fusion and also myotube size and length. Syndecan-4 expression has also been shown to influence the actin nanostructure of myotubes analyzed by superresolution dSTORM imaging, resulting in thicker cortical actin and also a reduced cell elasticity and thereby increased fusion efficiency. Beyond its role in physiological muscle differentiation and fusion, syndecan-4 expression exhibits alterations in human rhabdomyosarcoma samples. We detected syndecan-4 copy-number amplification in 28% of FNRMS cases accompanied by high levels of syndecan-4 expression suggesting that syndecan-4 can serve as a tumor driver gene in promoting rabdomyosarcoma development. During muscle differentiation, the gradually decreasing expression of syndecan-4 allows the activation of Rac1, thereby mediating myoblast fusion. However, high syndecan-4 expression inhibits myogenesis and promotes oncogenesis. Therefore, our findings shed light on the essential role of syndecan-4 in muscle differentiation and tumorigenesis.

## Materials and methods

### Cell culture and plasmids

C2C12 mouse myoblasts (ATCC; Massanas, VA, USA) were stably transfected with plasmids (OriGene Technologies Inc., TR513122, Rockville, MD, USA) expressing shRNAs (short hairpin RNAs) specific for syndecan-4 (shSDC4#1, target sequence: 5ʹ GAA CTG GAA GAG AAT GAG GTC ATT CCT AA 3ʹ; and shSDC4#2, target sequence: 5ʹ GCG GCG TGG TAG GCA TCC TCT TTG CCG TT 3ʹ) or a scrambled target sequence (5ʹ GCA CTA CCA GAG CTA ACT CAG ATA GTA CT 3ʹ) by X-tremeGENE (Roche; Basel, Switzerland) transfection reagent. Non-transfected cells were cultured in 80% DMEM (containing 4.5 g/L glucose, l-glutamine, and pyruvate; Lonza, Basel, Switzerland), 20% fetal bovine serum (FBS; Gibco, Life Technologies, Waltham, MA, USA), and 50 µg/mL gentamicin. The transfected cells were selected in a medium containing puromycin (4 µg/mL; InvivoGen, San Diego, CA, USA). For differentiation, an equal number of cells was seeded into six-well plates (1.8 × 10^5^ cells/well) for 24 h in growth medium, and then, differentiation was induced by shifting the cells into differentiation medium containing 2% horse serum (Gibco/Life Technologies, New Zealand).

RD (ATCC CCL-136) human rhabdomyosarcoma cells were obtained from ATCC (Massanas, VA, USA) and maintained in 90% DMEM (containing 4.5 g/L glucose, l-glutamine, and pyruvate; Lonza), 10% FBS (Gibco), and 50 µg/mL gentamicin.

### Animal model

To induce regeneration of the soleus muscle of male Wistar rats (weighing 300–320 g), the snake venom notexin (from *Notechis scutatus scutatus*; Sigma‐Aldrich, St. Louis, MO, USA) was injected along the entire length of the muscle (20 μg notexin in 200 μL of 0.9% NaCl) under chloral hydrate anesthesia as described previously [23]. The muscles were removed under anesthesia on days 0, 1, 3, 4, 5, 7, 10, and 14 after injury (*n* = 4 in each group). All animal experiments were conducted with approval obtained from the Animal Health Care and Control Institute, Csongrad County, Hungary.

### Gel electrophoresis and immunoblotting

Cells were lysed in RIPA buffer [20 mM Tris–HCl (pH 7.5), 150 mM NaCl, 1 mM Na_2_EDTA, 1 mM EGTA, 1% NP-40, 1% sodium deoxycholate, 2.5 mM sodium pyrophosphate, 1 mM b-glycerophosphate, 1 mM Na_3_VO_4_, 1 μg/mL leupeptin; Cell Signaling Technology, #9806], supplemented with 1 mM NaF (Sigma-Aldrich, St. Louis, MO, USA) and protease inhibitor cocktail (Sigma-Aldrich). Samples were centrifuged at 13,000 rpm for 5 min at 4 °C to eliminate cellular debris. Soleus muscles were homogenized in a buffer containing 50 mM Tris–HCl pH 7.6, 100 mM NaCl, 10 mM EDTA, 1 mM NaF, 1 mM Na_3_VO_4_, and protease inhibitor cocktail (Sigma‐Aldrich) and then centrifuged at 13,000 rpm for 5 min at 4 °C to remove the pellet.

Protein concentration in the samples was determined using a BCA protein assay kit (Pierce Chemical, Rockford, IL, USA), and equal amounts of proteins were resolved on polyacrylamide gel and transferred onto Protran membranes (GE Healthcare Amersham™, Little Chalfont, UK). Membranes were incubated with the following antibodies: rabbit monoclonal anti-cofilin (D3F9, #5175), anti-phospho-cofilin(Ser3) (77G2, #3313), mouse monoclonal anti-GAPDH (#2118), rabbit polyclonal anti-PAK1 (#2602), phospho-PAK1(Thr423) (#2601) [all obtained from Cell Signaling Technology (Danvers, MA, USA)], rabbit polyclonal anti-desmin (DAKO, M076029-2; Agilent, Santa Clara, CA, USA), anti-MyoD (c-20) (Santa Cruz, sc-377460, Dallas, TX, USA), anti-MyoG (Sigma-Aldrich, MAB3876), and anti-SDC4 (Santa Cruz, sc-9499). After incubation with the appropriate horseradish peroxidase-conjugated anti-IgG secondary antibodies [anti-mouse (P0161) and anti-rabbit (P0448)] from DAKO (Glostrup, Denmark), the peroxidase activity was visualized using the enhanced chemiluminescence procedure (Advansta, Menlo Park, CA, USA). Signal intensities were quantified using the QuantityOne software program (Bio‐Rad, Hercules, CA, USA).

### Rac1 activation assay

Approximately, 70–80% of confluent cell cultures were lysed with Mg^2+^ lysis buffer (Merck, Darmstadt, Germany) containing 25 mM HEPES, pH 7.5, 150 mM NaCl, 1% Igepal CA-630, 10 mM MgCl_2_, 1 mM EDTA, and 2% glycerol and supplemented with 1 mM NaF (Sigma-Aldrich), 1 mM Na_3_VO_4_ (Sigma-Aldrich), and protease inhibitor cocktail (Sigma-Aldrich). Then, the lysates were centrifuged (14,000×*g* for 5 min at 4 °C), the supernatant was aspirated, and then the pellet was removed. For the detection of active Rac1-GTP, the Rac1 Activation Magnetic Beads Pull-down Assay (Merck, 17_10393, Darmstadt, Germany) was applied according to the manufacturer’s instructions. In the samples, Rac1-GTP was bound to the p21-binding domain (PBD) of the Rac1-effector p21-activated kinase (PAK1) fused to the magnetic beads. Briefly, a reaction mixture of 10 µg of magnetic beads per 0.5 mL of cell lysates was incubated for 45 min at 4 °C with gentle stirring, after which the beads were washed and resuspended in 2 × Laemmli reduction sample buffer and boiled for 5 min. Then, the samples were applied to a polyacrylamide gel along with the beads and transferred onto Protran nitrocellulose membranes (GE Healthcare Amersham™). The membranes were first incubated with anti-Rac1 antibody (clone 23A8, Merck; 05-389, Darmstadt, Germany) and then with the appropriate HRP-conjugated secondary antibody (goat anti-mouse, DAKO, P0161).

### Rac1 GTPase inhibition

Rac1 activity was inhibited using NSC23766 trihydrochloride (Sigma-Aldrich) during myoblast differentiation. Cells were seeded into six-well plates (1.8 × 10^5^ cells/well) in growth medium and then shifted to a differentiation medium containing 50 μM NSC23766, and the medium was changed every 2 days.

### Fluorescence staining

For desmin immunostaining, myotubes were fixed with 4% paraformaldehyde on the 5th day of differentiation, and after 5-min permeabilization with 0.1% Triton X-100 in PBS, the samples were blocked in 0.1% bovine serum albumin (BSA) in PBS. For staining the differentiated myotubes, the samples were incubated overnight with mouse anti-desmin (Biocare Medical, 901-036-081214, Pacheco, CA, USA) primary antibody at 4 °C, followed by incubation with anti-mouse Alexa Fluor 488-conjugated secondary antibody (Jackson Immunoresearch, West Baltimore Pike, West Grove, PA, USA) for 20 min. Nuclei were stained with Hoechst 33258 (Sigma-Aldrich), and samples were coated with a fluorescent mounting medium (DAKO).

For visualization of actin filaments, the myotubes were fixed with 4% paraformaldehyde and incubated with PBS containing 0.9% Triton X-100 and 4% BSA for 30 min. Then, the samples were labeled with Alexa-647-conjugated phalloidin (Cell Signaling, #8878S). Following nuclear staining with Hoechst 33258 (Sigma-Aldrich), the samples were immediately processed for dSTORM and confocal imaging.

### Myotube analysis

Widefield fluorescence images of desmin- and Hoechst 33258-stained samples were acquired using a Nikon Eclipse Ni-U fluorescence microscope (Nikon Instruments Inc., Melville, NY, USA) with a 10 × objective lens (Nikon FI Plan Fluor 10 ×, DIC N2, NA = 0.30) and analyzed using the Digimizer image analysis software (MedCalc Software, Belgium). A total of 16–18 fields of view per three independent experiments were analyzed in each cell line. The differentiation index was derived as the ratio of the number of desmin-positive cells and total number of nuclei. The value of fusion index was obtained by dividing the number of nuclei belonging to the desmine-positive myotubes with all counted nuclei. The area and length of each myotube were also quantified.

### Confocal laser scanning microscopy

Confocal images were captured using a Nikon C2 + confocal scan head attached to a Nikon Eclipse Ti-E microscope. Confocal and superresolved dSTORM images were captured sequentially using the same microscope objective (Nikon CFI Apochromat TIRF, NA = 1.49, × 100) throughout the experiments to minimize spatial drift and reduce image registration issues. The setup and data acquisition process were controlled using the Nikon NIS-Elements 5.02 software, and the captured images were postprocessed in ImageJ-Fiji (https://fiji.sc/). The Nikon Laser Unit was used to set the wavelengths and the power of the applied lasers operated at 405 and 647 nm.

### dSTORM measurements

Superresolution direct stochastic optical reconstruction microscopy (dSTORM) measurements were performed on a custom-made inverted microscope based on a Nikon Eclipse Ti-E frame. EPI-fluorescence illumination was applied at an excitation wavelength of 647 nm (2RU-VFL-P-300-647-B1, Pmax = 300 mW, MPB Communications Ltd). The laser intensity was set to 2–4 kW/cm^2^ on the sample plane and controlled using an acousto-optic tunable filter. An additional laser (405 nm, Pmax = 60 mW; Nichia) was used for reactivation. A filter set from Semrock (Di03-R405/488/561/635-t1-25x36 BrightLine^®^ quad-edge superresolution/TIRF dichroic beamsplitter, FF01-446/523/600/677-25 BrightLine^®^ quad-band bandpass filter, and an additional AHF 690/70 H emission filter) was inserted into the microscope to spectrally separate the excitation and emission lights. The images of individual fluorescent dye molecules were captured using an Andor iXon3 897 BV EMCCD camera (512 × 512 pixels with 16-μm pixel size) with the following acquisition parameters: exposure time = 30 ms, EM gain = 200, and temperature = − 75 °C. Typically 20,000–50,000 frames were captured from a single ROI. During the measurement, the Nikon Perfect Focus System maintained the sample in focus. High-resolution images were reconstructed using the rainSTORM localization software [41]. The mechanical drift introduced by either the mechanical movement or thermal effects was analyzed and reduced using an autocorrelation-based blind drift correction algorithm.

### dSTORM buffer

dSTORM experiments were conducted in a GLOX switching buffer [42], and the sample was mounted onto a microscope slide. The imaging buffer was an aqueous solution diluted in PBS containing an enzymatic oxygen scavenging system, GluOx [2000 U/mL glucose oxidase (Sigma-Aldrich, G2133-50KU), 40,000 U/mL catalase (Sigma-Aldrich, C100), 25 mM potassium chloride (Sigma-Aldrich, 204439), 22 mM tris(hydroxymethyl)aminomethane (Sigma-Aldrich, T5941), and 4 mM tris(2-carboxyethyl)phosphine (TCEP) (Sigma-Aldrich, C4706)] with 4% (w/v) glucose (Sigma-Aldrich, 49139) and 100 mM β-mercaptoethylamine (MEA) (Sigma-Aldrich, M6500). The final pH was set to 7.4.

### Cortical actin bundle width measurements

The localization information of the selected structures was exported by the rainSTORM program using the “Export box section” tool into the IFM Analyzer code written in MATLAB R2018b. The IFM Analyzer code was originally developed for the quantitative evaluation of dSTORM images on Indirect Flight Muscle Sarcomeres. The same code was used in the present study to retrieve the epitope distribution information from raw localization data and determine the width of the cortical actin bundles.

First, a straight line was roughly fitted on the localization coordinates in order to determine the orientation of the selected bundle. A Gaussian kernel (with a kernel size of 40–80 nm, depending on the localization density) was applied to obtain a smoothed localization density map. Then, a polynomial was fitted along the maxima of the localization density map, considering the curvature of the selected actin bundles. The distance of each localized point from the fitted curve was determined numerically and depicted in a histogram.

The histograms were fitted with a single Gaussian curve, and the localization precision [43] was used to deconvolve these distributions. The linker length was set to 0 nm due to the small size of phalloidin [44]. The measured FWHM of these distribution profiles was considered to be the width of the actin bundles.

### Skeletonization

An additional MATLAB code was written to skeletonize the superresolution images and determine the number and length of branches of the actin filaments. First, the images were binarized with a threshold gain of Otsu’s method [45] or with a threshold set manually through ImageJ-Fiji. The images were filtered with a 2D Gaussian smoothing kernel with a standard deviation of 3–4 pixels (60–80 nm) to homogenize the pixelated images and were again binarized using the Otsu’s method. Built-in MATLAB functions (bwskel) were used to skeletonize the binary images and to calculate the branch numbers and branch lengths (bwmorph and bwdistgeodesic). Short branches were omitted from the calculation (the minimum branch size was set to 120 nm).

### Atomic force microscopy

Cells (all types) were cultured on the surface of a glass coverslip. After medium change, the coverslips were mounted into the heating chamber of the microscope in a standard glass-bottomed plastic Petri dish and maintained at 37 °C during measurements. Elastic maps were recorded using an NTegra Spectra (NT-MDT Spectrum Instruments, Moscow, Russia) atomic force microscope running the Nova Px 3.4.1 driving software, mounted on the top of an IX73 inverted optical microscope (Olympus, Shinjuku, Tokyo, Japan) to facilitate initial positioning. Elastic maps were recorded in Hybrid mode of the instrument using a loading force of < 0.5 nN and a repetition rate of 200–400 Hz, achieving a resolution of < 100 nm for adjacent force curves. For experiments, 60-µm-long overall gold-coated cantilevers with a V-shaped tip were used (OBL10, Bruker). Each cantilever was calibrated before the experiments based on the Sader method [46]. Elastic parameters were calculated using the Hertz model with the assistance of the driving software.

### Rhabdomyosarcoma cases and genomic datasets

Genomic data from 199 specimens, collected from 199 patients and deidentified before use, were compiled from the following three dataset sources: the National Cancer Institute, the Children’s Oncology Group, and the University of Texas Southwestern (UTSW). Genomics analyses of archived patient samples were conducted at the UTSW Medical Center with the approval of its institutional review board (STU 102011-034). The original genomic data is deposited to dbGAP database with accession number phs000720.

### Genomic sequencing, copy number, and gene expression data analysis

Whole-genome and whole-exome sequencing reads were aligned to the human reference genome (hg19), and somatic protein-altering mutations were identified using the Genome Analysis Tool Kit pipeline. SNP arrays were processed using the SNP-FASST segmentation algorithm implemented in the Nexus BioDiscovery software (BioDiscoveryEl Segunda, CA, USA). Significantly altered CNVs were examined using the GISTIC method using a default q value of 0.25 to define statistical significance, as described previously [47]. For gene expression data, RNA was processed using the Affymetrix Exon 1.0 ST array platform according to the manufacturer’s recommendations (Affymetrix, CA, USA). CEL files were analyzed using R/BioConductor with robust multiarray average normalization and custom PERL scripts as described earlier [48].

### Statistical analysis

Statistical analyses were conducted using the GraphPad Prism 6 software (GraphPad Software Inc., San Diego, CA, USA), Student’s *t* test and one-way ANOVA, and a post hoc test (Sidak) for peer pair comparison. All evaluated data were expressed as average + SEM. *p* < 0.05 denoted statistical significance.

## Results

### Syndecan-4 knockdown increases myoblast differentiation and fusion in vitro

**Fig. 1:**
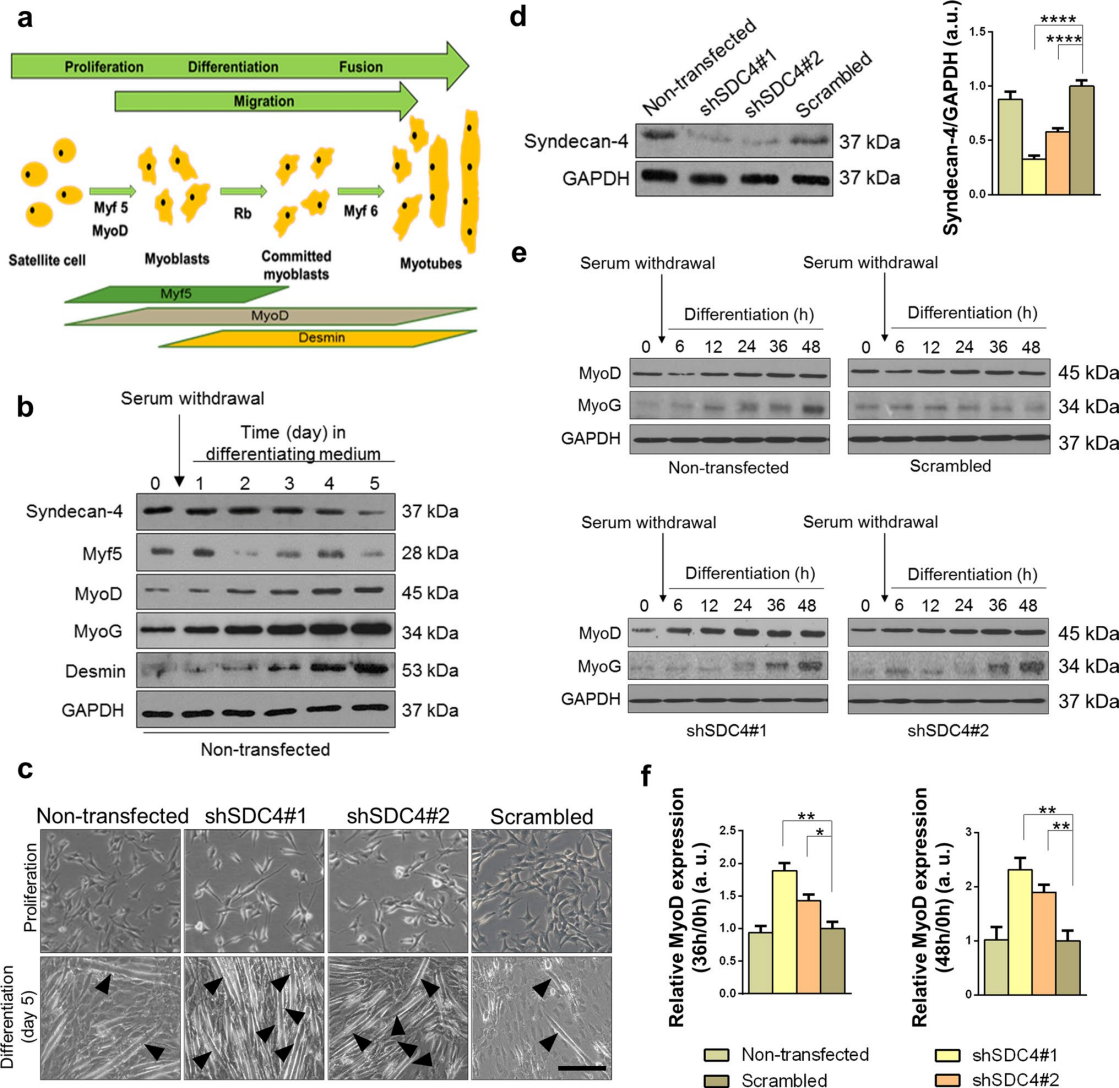
Effect of silencing syndecan-4 expression on C2C12 myoblasts. **a** Schematic summary of muscle regeneration. Myoblasts proliferate, differentiate, and fuse to form multinucleated myotubes. **b** Protein extracts of C2C12 murine myoblasts were harvested at indicated time points of differentiation and subjected to SDS/PAGE. Representative immunoblots depict the expression levels of syndecan-4, Myf5, MyoD, MyoG, and desmin during differentiation. GAPDH was used as the loading control. **c** C2C12 cells were stably transfected with shRNA to decrease the expression of syndecan-4 (shSDC4#1 and shSDC4#2) or a scrambled sequence. Representative phase-contrast images show the phenotype of cell lines. Arrowheads indicate the myotubes. Bar: 100 µm. **d** Representative western blot experiment shows the level of syndecan-4 in the different cell lines. Quantification of the results is shown, *n* = 7 independent experiments, mean + SEM; **p < 0.01; *p < 0.05. **e** MyoD and MyoG expression in the cell lines was monitored during differentiation for 48 h. Representative western blot results show MyoD and MyoG expression at indicated time points. GAPDH represents the equal loading of samples. Quantification of results is reported, *n* = 3 independent experiments, mean + SEM; ***p < 0.001; **p < 0.01; *p < 0.05

Skeletal muscle is constantly renewed in response to injury, exercise, or muscle diseases. The satellite cells are quiescent in the healthy muscle; they are stimulated by local damage to proliferate extensively and form myoblasts that will subsequently migrate, differentiate, and fuse to form muscle fibers (Fig. 1a).

The expression of syndecan-4 gradually decreased during the 5-day differentiation of C2C12 murine myoblasts, and the proliferating myoblasts showed higher syndecan-4 levels, whereas the differentiated myotubes showed lower syndecan-4 levels (Fig. 1b). To monitor the process of myoblast differentiation, we evaluated the amount of three myogenic transcription factors, Myf5, MyoD, MyoG, and desmin, a muscle-specific intermediate filament. The expression of Myf5 showed a peak at day 1, whereas those of MyoD, MyoG, and desmin continuously increased, indicating the appropriate differentiation of the samples.

To analyze whether syndecan-4 participates in myoblast differentiation in vitro, we reduced the expression of syndecan-4 by shRNA-mediated silencing in C2C12 cells. Two shRNA constructs targeting syndecan-4 were used, shSDC4#1 and shSDC4#2, respectively. Silencing the expression of syndecan-4 caused alterations in the morphology of cells, wherein the shape of cells was elongated in the growth medium (Fig. 1c). Syndecan-4 expression in the cell lines was checked by western blotting, which revealed more reduction in shSDC4#1 cells than in shSDC4#2 cells. Transfection with shRNA carrying the scrambled sequence exhibited no effect on syndecan-4 expression in the cells (Fig. 1d).

We induced the differentiation of cell lines at 90% confluence by replacing the growth medium with differentiation medium for 5 days. Representative phase-contrast images depicted the differentiated cultures, wherein the myotubes were clearly formed at day 5 (Fig. 1c). Next, we monitored myoblast differentiation for 48 h and evaluated the changes in MyoD and MyoG expression. Representative immunoblots showed that both MyoD and MyoG expression increased earlier in syndecan-4 silenced cells during differentiation (Fig. 1e). Among the examined time points, we observed a significantly greater increase in MyoD expression at 36 and 48 h of differentiation in both syndecan-4-silenced cell cultures (Fig. 1f), indicating the enhanced differentiation ability of these cell lines.

**Fig. 2:**
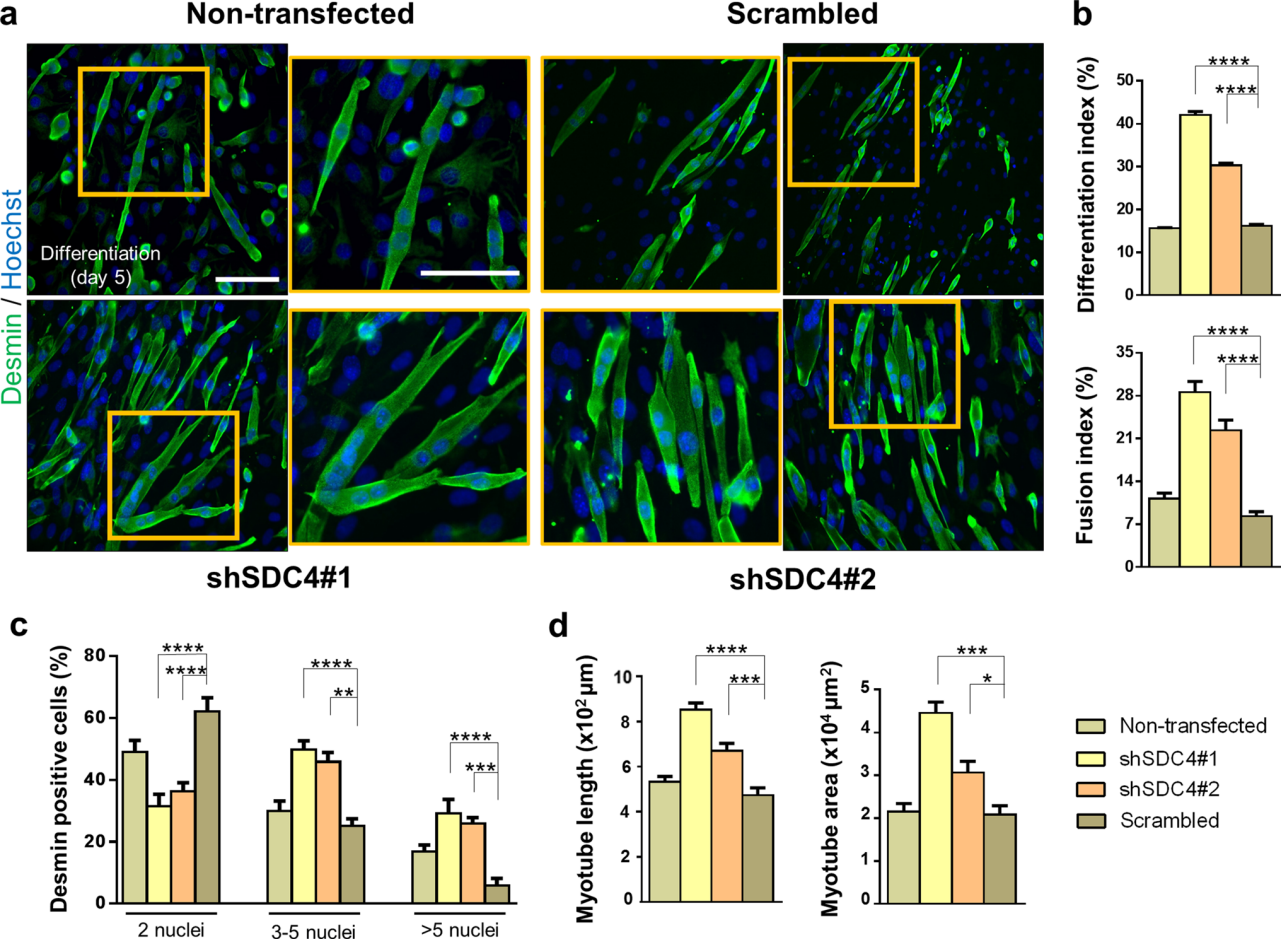
Silencing syndecan-4 expression enhances the fusion of myoblasts. **a** Representative anti-desmin-stained (Alexa Fluor 488, green) images depict the myotube formation of the non-transfected, scrambled, and syndecan-4 silenced (shSDC4#1 and shSDC4#2) cell lines. The indicated regions are shown in higher magnification. Nuclei were stained with Hoechst 33258 (blue). Bar: 100 µm. **b** Quantification of the differentiation index (number of desmin-positive cells/total number of nuclei) and fusion index (number of nuclei in myotubes/total number of nuclei) of the cell lines. **c** Numbers of nuclei in desmin-positive myotubes after 5 days of differentiation. **d** Myotube length and myotube area of the different cell lines. 16–18 fields of view per cell line were analyzed; *n* = 3 independent experiments; mean + SEM; ****p < 0.0001; ***p < 0.001; **p < 0.01; *p < 0.05

To further analyze the role of syndecan-4 in mammalian myogenesis, we evaluated myotube formation after 5-day differentiation. Desmin-stained representative images depicted differences in the number and shape of myotubes after silencing syndecan-4 expression, wherein syndecan-4-knockdown cells formed much longer and bulkier myotubes than those of control cell lines (Fig. 2a). We calculated the differentiation index by expressing the number of desmin-positive cells as a percentage of total number of nuclei and the fusion index by expressing the number of myonuclei within desmin-positive myotubes with ≥ 2 nuclei as a percentage of total nuclei of the analyzed sample. We found significant increases in the differentiation index and fusion index in both syndecan-4 silenced cell lines (Fig. 2b). Nuclear number analysis revealed that the number of nuclei in the myotubes increased significantly after syndecan-4 knockdown. The majority of syndecan-4 silenced myotubes contained 3–5 or > 5 nuclei, whereas control cell lines contained primarily 2 nuclei per myotube (Fig. 2c), suggesting that syndecan-4 knockdown is involved in myonuclear accretion to promote myotube formation. Moreover, both the area and length of myotubes were larger in syndecan-4 silenced cell lines (Fig. 2b).

Interestingly, following the overexpression of syndecan-4, myotube formation was not observed in C2C12 cells (Supplementary Fig. 1a), and the levels of both MyoD and MyoG decreased (Supplementary Fig. 1b) suggesting the decreased differentiation of myoblasts. Moreover, the levels of cyclin E and cyclin D increased, while p21 expression decreased (Supplementary Fig. 1b), indicating the enhanced transition of the G1/S phases of the cell cycle in these cells.

### Rac1 activity is required for increased fusion of syndecan-4-knockdown cells

**Fig. 3:**
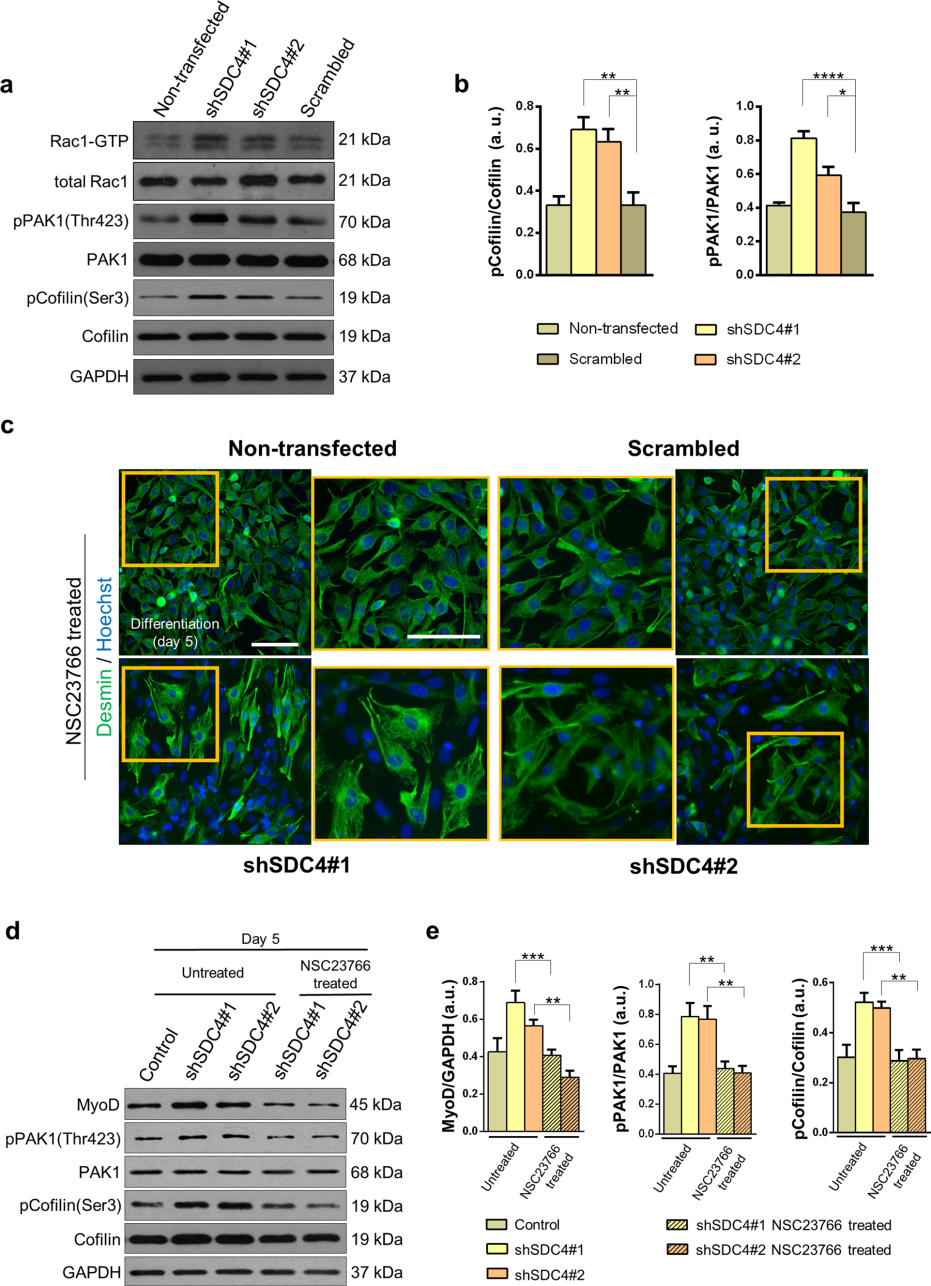
Changes in Rac1-GTP, phospho-Pak1(Thr423), and phospho-cofilin(Ser3) levels of myoblasts after silencing syndecan-4 expression. **a** Representative western blot results depict changes in the amount of active Rac1 (Rac1-GTP), phospho-PAK1(Thr423), and phospho-cofilin(Ser3) levels in the different cell lines grown in proliferation medium. GAPDH shows the equal loading of samples. **b** Quantification of the effect of syndecan-4 silencing on cofilin and PAK1 phosphorylation. **c** Activation of Rac1 was inhibited by NSC23766 (50 µM), and cells were differentiated for 5 days. Representative wide field fluorescence images were acquired on the 5th day of differentiation (green: desmin; blue: Hoechst) of NSC23766-treated cells. The indicated regions are shown in higher magnification. Bar: 100 µm. **d** Representative immunoblots show MyoD, phospho-PAK1(Thr423), PAK1, phospho-cofilin(Ser3), and cofilin levels in differentiated cell cultures with or without NSC23766 treatment. GAPDH indicates the equal loading of samples. Quantification of results is shown in panel **e**, *n* = 3 independent experiments, mean + SEM; ***p < 0.001; **p < 0.01; *p < 0.05

Because the activation of Rac1 GTPase increases myoblast fusion [12], Rac1 is necessary and sufficient for rhabdomyosarcoma cell migration and invasion [49], and syndecan-4 regulates Rac1 level [33, 34], we next analyzed the role of Rac1 in syndecan-4-dependent myoblast differentiation and fusion. First, we monitored Rac1-GTP levels in the proliferating cells using a pull-down assay with the p21-binding domain of PAK1. Our results indicated that silencing syndecan-4 expression increased the amount of Rac1-GTP (Fig. 3a). We also performed western blot analysis to examine whether silencing the expression of syndecan-4 affected the phosphorylation of the Rac1-effector PAK1/cofilin signaling. PAK1 is a Ser/Thr kinase regulated by, among others, Rac1, and regulates LIMK1/cofilin activity and consequently the remodeling of the actin cytoskeleton. We observed that both the phospho-PAK1(Thr423)/PAK1 and phospho-cofilin(Ser3)/cofilin ratios were elevated in syndecan-4 knockdown cells (Fig. 3a, b).

As syndecan-4 knockdown increased the Rac1-GTP level and the phosphorylation of PAK1 and cofilin, we next tested the effect of Rac1 inhibition on myoblast differentiation after silencing syndecan-4 expression. During differentiation, myoblasts were treated with NSC23766, a specific Rac1 inhibitor. Representative desmin-stained widefield fluorescence images depicted that NSC23766 treatment inhibited myotube formation in either control or silenced cells, although desmin was expressed (Fig. 3c). Moreover, NSC23766 administration abrogated the increases in MyoD expression and also the increases in pPAK1(Thr423)/PAK1 and phospho-cofilin(Ser3)/cofilin ratios in syndecan-4 silenced cells (Fig. 3d, e).

### The levels of Tiam1, phospho-PAK1, and phospho-cofilin are gradually reduced during in vitro and in vivo myogenesis 

**Fig. 4:**
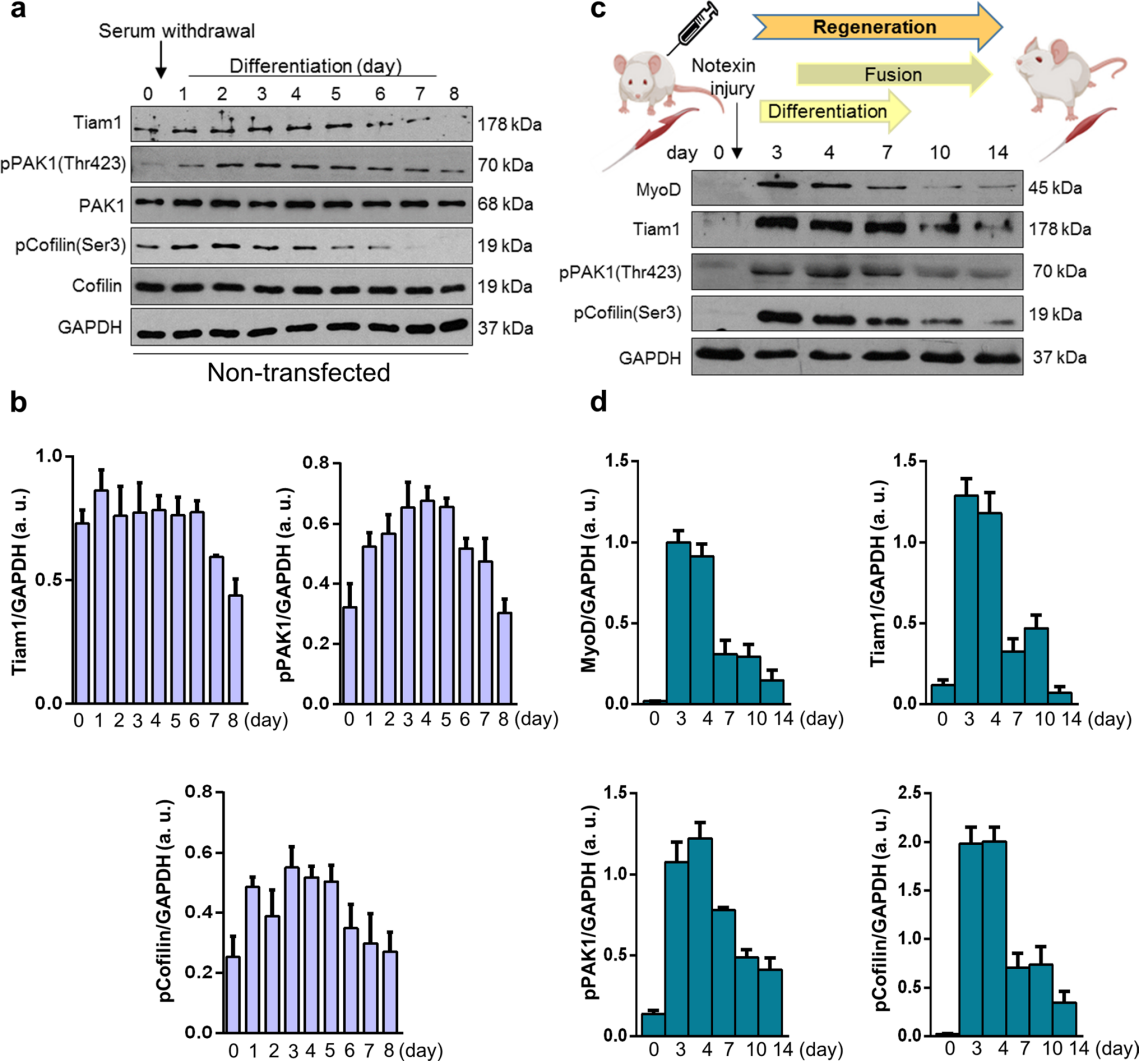
Changes in Tiam1, phospho-Pak1(Thr423), and phospho-cofilin(Ser3) levels during in vitro myoblast differentiation and in vivo muscle regeneration. **a** Representative western blot results show Tiam1, phospho-Pak1(Thr423), Pak1, phospho-cofilin(Ser3), and cofilin levels at indicated time points of the differentiation of non-transfected C2C12 myoblasts. GAPDH represents the equal loading of samples. Quantification of results is shown in panel **b** *n* = 4 independent experiments, mean + SEM. **c** Representative results of western blot experiments depict changes in MyoD, Tiam1, phospho-Pak1(Thr423), and phospho-cofilin(Ser3) levels during the in vivo regeneration of the soleus muscle of rat after notexin-induced necrosis. **d** Quantification of results of M. soleus samples is shown, *n* = 3 independent experiments, mean + SEM

Tiam1 is a GEF mediating GTP binding and thereby the activation of Rac1. Because Rac1-GTP level increases during myoblast fusion, we next investigated the changes in Tiam1 levels during in vitro myoblast differentiation and in vivo skeletal muscle regeneration. During the 8-day differentiation period of C2C12 cells, the high Tiam1 level continuously decreased after the 5th day. We also evaluated the amounts of Rac1-effector phospho-PAK1 and phospho-cofilin and observed that during the early stages of differentiation, from day 2 onward, an intense increase occurred followed by a decrease from day 5 in phospho-PAK1 (Thr423) levels (Fig. 4a, b). Consistent with phospho-PAK1 levels, the levels of phospho-cofilin(Ser3) exhibited the same pattern (Fig. 4a, b).

To monitor the levels of proteins during in vivo skeletal muscle regeneration, muscle regeneration was induced by injecting the snake venom notexin, which induces necrosis in the soleus muscle of the rat but retains the function of the satellite cells of the muscle. After the skeletal muscle damage, regeneration begins with the activation of resting satellite cells, followed by proliferation and fusion, and finally the formation of a healthy, functional muscle. In this model system, by day 4 post injury, regenerating small-caliber myofibers are formed, and by day 14, the muscle almost restores the normal morphology with the presence of centrally located nuclei and an increased interstitial space between the muscle fibers [23]. The regeneration process was well illustrated by the changes in MyoD level as it was increased after the injury and almost reached the baseline, i.e., physiological state at day 14 postinjury (Fig. 4c). The levels of Tiam1, phospho-PAK1, and phospho-cofilin were also evaluated in soleus muscle samples at different days postinjury to monitor the changes during regeneration (Fig. 4c). We found remarkable increases in the levels of all the examined molecules at days 3 and 4 postinjury, which then gradually decreased and finally reached the initial state (Fig. 4c, d).

To summarize, during both in vitro differentiation and in vivo skeletal muscle regeneration, the levels of Rac1 activator Tiam1 and the phosphorylation of the Rac1-effector PAK1 and cofilin were transiently increased. These increases can result in an intense remodeling of the actin network during the formation of myotubes. However, during in vivo experiments, the observed changes may originate from other cell types (e.g., macrophages) of the regenerating muscle beside muscle cells/fibers.

### Silencing syndecan-4 expression affects the nanoscale structure of the actin network by increasing cortical actin thickness and number of branches

Differentiation and fusion require changes in the cytoskeletal elements of the cell, rearrangement of the actin cytoskeleton, and cell–matrix connections. Syndecan-4 establishes contact with the actin cytoskeleton, as its cytoplasmic domain binds to alpha-actinin, a cross-linking protein between actin filaments [31]. Furthermore, in this study, we showed that syndecan-4 affects the activity of Rac1 in myoblasts, a key regulator of actin remodeling. Considering these important roles of syndecan-4 in actin cytoskeleton organization, we monitored the changes in the actin nanostructure during differentiation after silencing syndecan-4 expression.

**Fig. 5:**
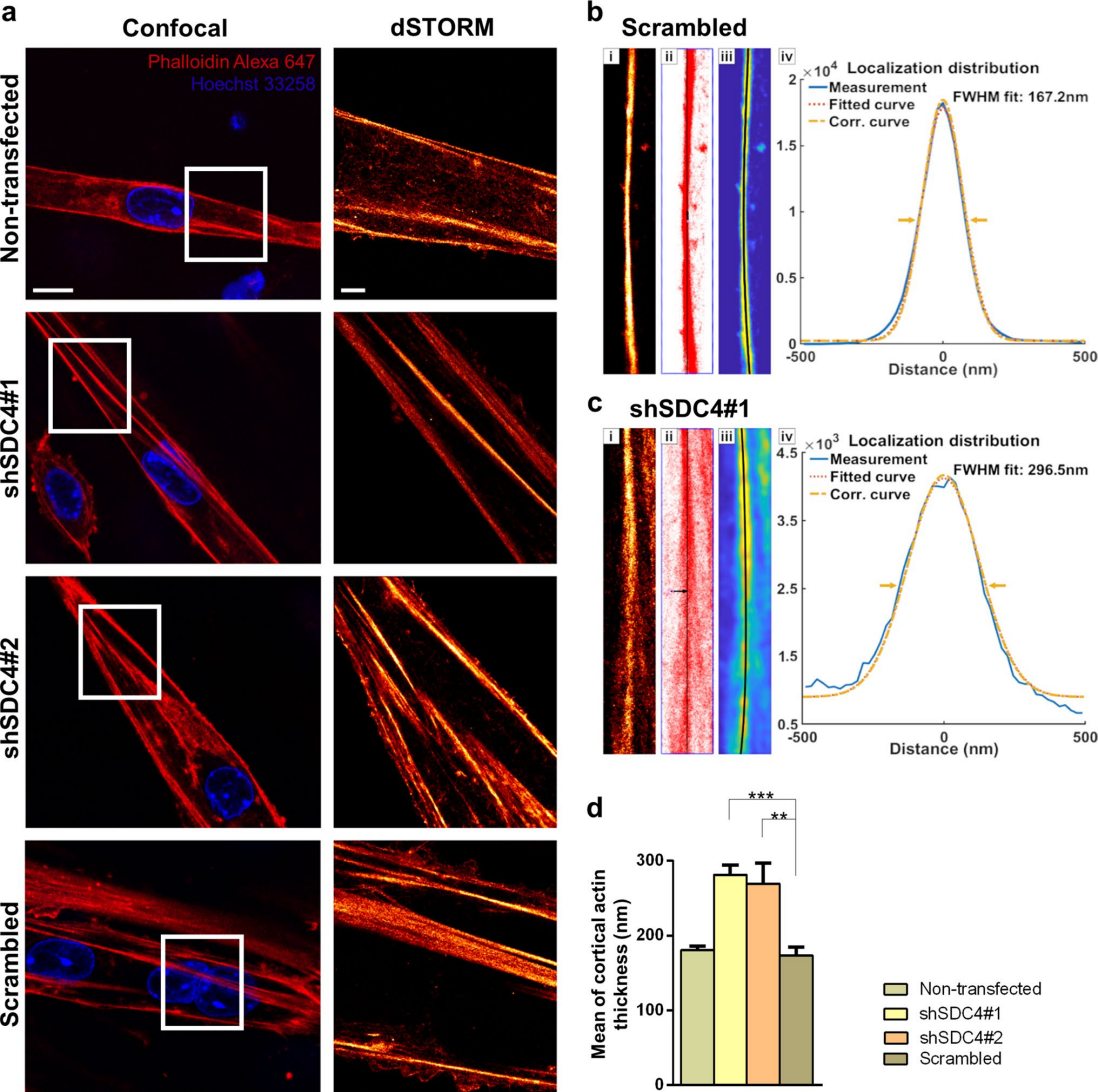
Examination of cortical actin thickness in myotubes using dSTORM superresolution microscopy. **a** Representative two-color confocal and single-color dSTORM fluorescence images of non-transfected, scrambled, and syndecan-4 silenced cell lines after 3 days of differentiation. Actin and DNA were stained with Alexa Fluor™ 647 phalloidin (red) and Hoechst 33258 (blue), respectively. Panels **b** and **c** show the evaluation process and the results for a representative control and silenced pixelated dSTORM (**i**) images. After selection of the region of interest (**i**), all the individual localizations (red dots) were used to fit a line (black) to the actin bundles (**ii**). The resampled localization density maps (**iii**) were used to calculate and summarize the cross sections perpendicular to the bundles. The localization distributions of the measured, fitted, and corrected cross sections of the selected cortical actin bundles of the silenced and control samples are shown in panels (**iv**). The statistical evaluation for n = 12 independent experiments is shown (**d**); mean + SEM; ***p < 0.001; **p < 0.01). Scale bar: 10 µm (confocal images), 2 µm (dSTORM images)

We analyzed the actin filaments by confocal and single-molecule localization superresolution dSTORM microscopy after 3 days from the onset of differentiation (Fig. 5a). Remarkably, superresolution dSTORM imaging reveals the subdiffraction structure of the actin cytoskeleton and enables a more sophisticated experimental comparison of the cytoskeletal structure in the different cell lines. The reduced fluorescence background and enhanced resolution enable the visualization of the orientations and densities of individual actin bundles. For calculating the cortical actin bundle width, the raw localization data of dSTORM images were used, and based on a localization density map, the width of the actin bundles was determined. Representative recordings of a scrambled (Fig. 5b) and shSDC4#1 (Fig. 5c) cell and the evaluation method are shown in Fig. 5b, c. The histograms depict the distance of each localized point of the actin bundle from the fitted line (black lines in Fig. 5b, c). The measured data were fitted with a Gaussian distribution and a corrected curve was also calculated taking into consideration the localization precision. Due to the high precision of the accepted localizations (< 40 nm) the correction did not modify the original profile significantly. The measured full-width at half maximum (FWHM) of these distribution profiles was considered as the width of the actin bundles (Fig. 5b, c). Syndecan-4 silenced cell lines exhibited a significantly thicker cortical actin network than that of the control cells during differentiation, and the evaluation indicated an approximately 50% broadening of the silenced cell lines compared to that of the non-transfected and scrambled cell lines (Fig. 5d).

**Fig. 6:**
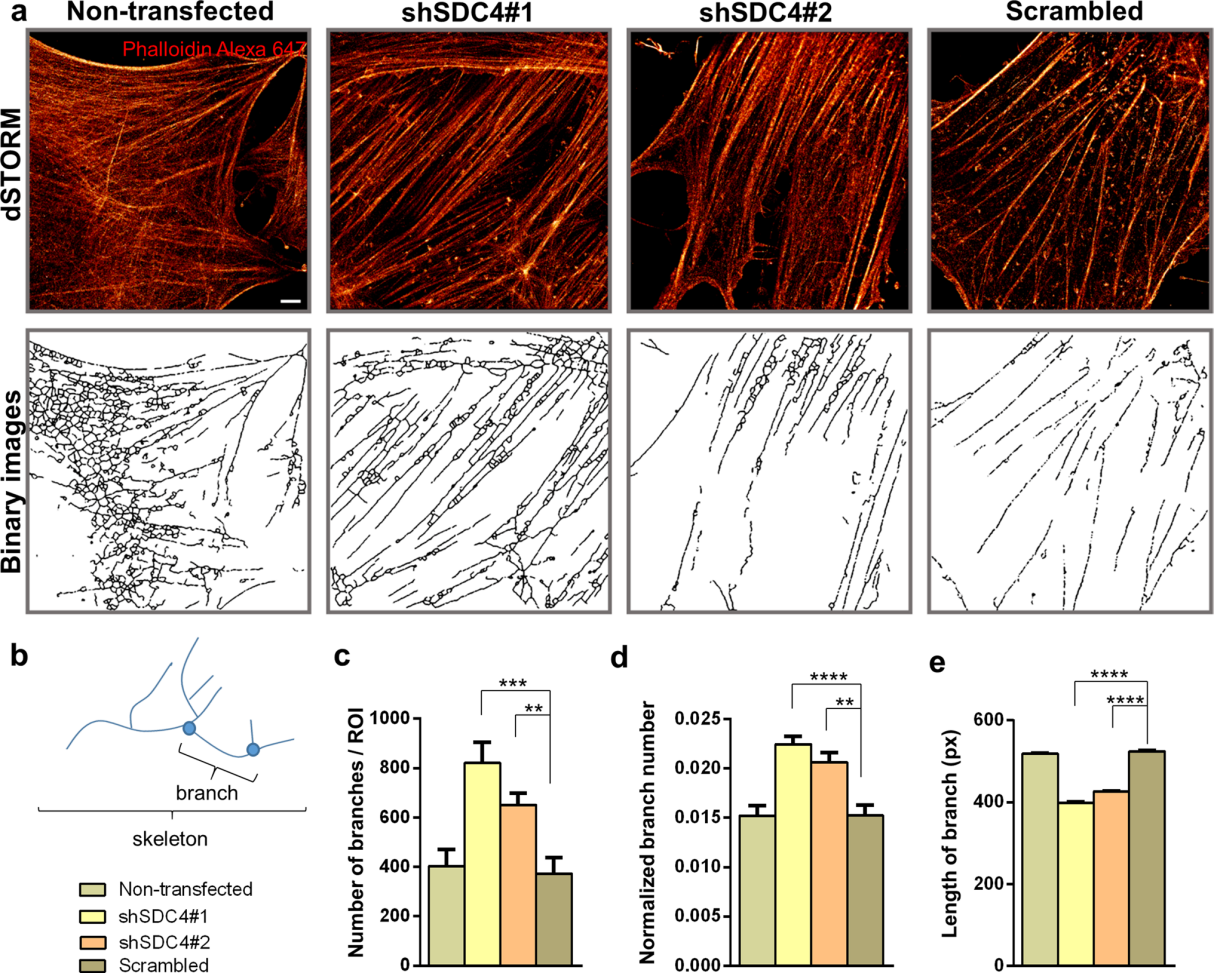
dSTORM analysis of the actin network of differentiated cells. **a** Phalloidin-stained (Alexa 647, red) representative dSTORM and skeletonized binary images of a non-transfected cell line, two syndecan-4 silenced (shSDC4#1 and shSDC4#2) cell lines, and a scrambled sample. Cells were differentiated for 3 days. **b** The primary structures of the actin cytoskeleton were divided into smaller branches terminated by branch points. The number of branches (**c**), the normalized branch number (**d**), and the length of branches (**e**) were used to quantify the four cell lines based on *n* = 6–12 independent experiments (mean + SEM; ****p < 0.0001; ***p < 0.001; **p < 0.01). Scale bar: 2 µm

For the nanoscale analysis of the branched structure of the actin network, the dSTORM images of 3-day-old mononuclear differentiated but not yet fused myoblasts were pixelizated and converted into binary images (Fig. 6a). Then, these skeletonized, binarized images were used for calculating the number and length of individual branches (Fig. 6b). The analysis revealed an increase in the number of branches and normalized branch number in syndecan-4 knockdown cells (Fig. 6c). The normalized branch number can be specified as the points (pixels) of the branch divided by all points of the skeleton, i.e., the amount of branching present in the skeleton, which implies another branch (Fig. 6d). However, the average length of the individual branches was shorter compared to that of control cells (Fig. 6e). These changes of the actin cytoskeleton can result in a more compact actin network that promotes fusion of the syndecan-4 silenced cells.

Next, we studied the effect of serum content of the media for the organization of actin nanostructure (Supplementary Fig. 2). Syndecan-4 silenced and control myoblasts were maintained in media containing either 20% FBS (proliferation media) or 2% horse serum (differentiation media), and the phalloidin-stained dSTORM images were binarized and analyzed. According to our results, the serum content of cell culture media (20% FBS vs. 2% horse serum) affected the actin nanostructure of the C2C12 cells (Supplementary Fig. 2a–c). By reducing the serum content, the length of individual branches of the actin cytoskeleton decreased in all cell lines (Supplementary Fig. 2c). Syndecan-4 silencing also decreased the length of branches independently of serum content (Supplementary Fig. 2c). The high serum content resulted in less brances of the actin nanoctructure in syndecan-4 silenced cells, while the number of branches of silenced cells increased in serum-reduced medium compared to controls (Supplementary Fig. 2c).

### Silencing syndecan-4 expression reduces the elasticity of myotubes

**Fig. 7:**
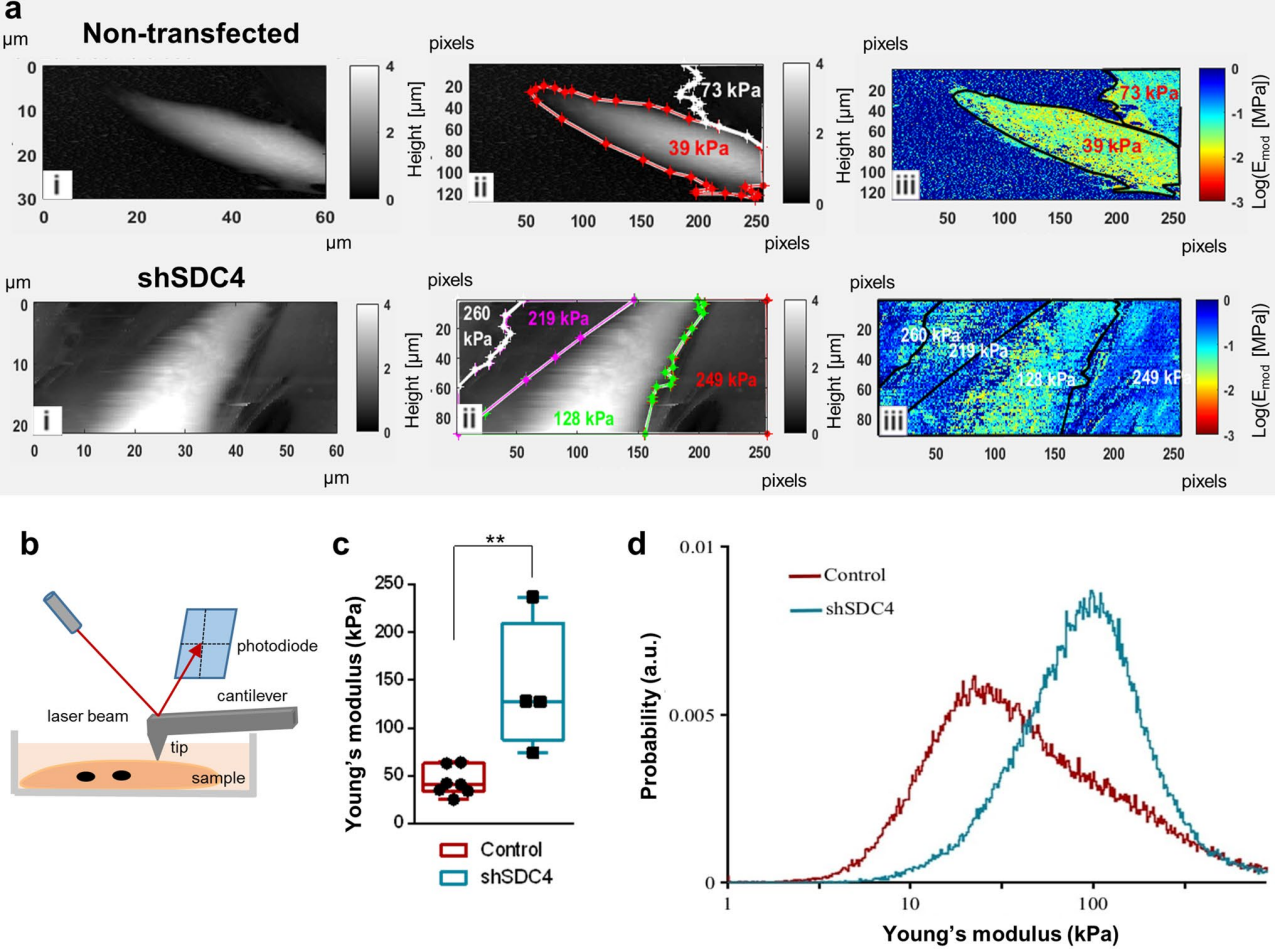
Atomic force microscopy studies revealed that syndecan-4-silenced cells have reduced elasticity. **a** Atomic force microscopy was performed after 3 days of differentiation, and representative images of non-transfected and syndecan-4 knockdown samples are shown. The first images (**i**) of control and syndecan-4-silenced cells show the height map of the sample. The white color shows cells that protrude from the dark underlay, and representative Young's modulus values are indicated (**ii**). In the elasticity maps (**iii**), the color encodes the Young's modulus (high modulus = hard, low modulus = soft). **b** Schematic illustration of atomic force microscope operation. **c** Box plots depict the Young's modulus values of syndecan-4 silenced and control cells. **d** Distribution of Young's modolus values of syndecan-4 silenced and control cells. Silencing of syndecan-4 expression decreased the flexibility of the cell. *n* = 7–5 independent experiments; **p < 0.01

Atomic force microscopy (AFM) allows capturing high-resolution 3D images while ensuring the optimal physical environment for the cells being examined. Some studies examined the morphology and transverse elasticity of myotubes in a rabbit and *Drosophila* model [50, 51]. The change in elasticity depends on the rearrangement of the cytoskeleton and the expression of the cytoskeletal actin–myosin protein [52]. Given the role of syndecan-4 in actin cytoskeleton remodeling, we hypothesized that syndecan-4 can affect the elasticity of cells. Therefore, we next examined how the elasticity of cells changes during fusion after silencing the expression of syndecan-4 (Fig. 7). AFM measurements were performed on myotubes at day 3 of differentiation (Fig. 7b). The grayscale images in Fig. 7a depict the height maps of the samples (control and syndecan-4 silenced cells), and the white color represents cells that protrude from the dark substrate. The pseudocolor images depict the Young's modulus (elastic modulus) of the samples (high modulus = hard, low modulus = soft; Fig. 7a). The color assignment to each pixel was based on the pixel intensity value, according to the calibration bar. These elasticity maps clearly reveal that the control cell encoded with yellow is softer than the blue substrate, whereas the hardness of the cell in the syndecan-4 knockdown cell line almost blends with that of the surrounding substrate. Therefore, silencing syndecan-4 expression decreases cell elasticity (Fig. 7c), i.e., these cells are tougher than control cells in accordance with the observed alterations in the cytoskeletal structure. Probability histograms calculated from all the obtained scans for control (dark red) and shSDC4 (light blue) cells are shown in Fig. 7d. Higher values on the X scale are associated with more rigid structures, whereas lower values are derived from softer material. Therefore, shSDC4 cells are predominantly harder than control cell.

### Copy-number amplification and increased expression of syndecan-4 in human rhabdomyosarcoma

**Fig. 8:**
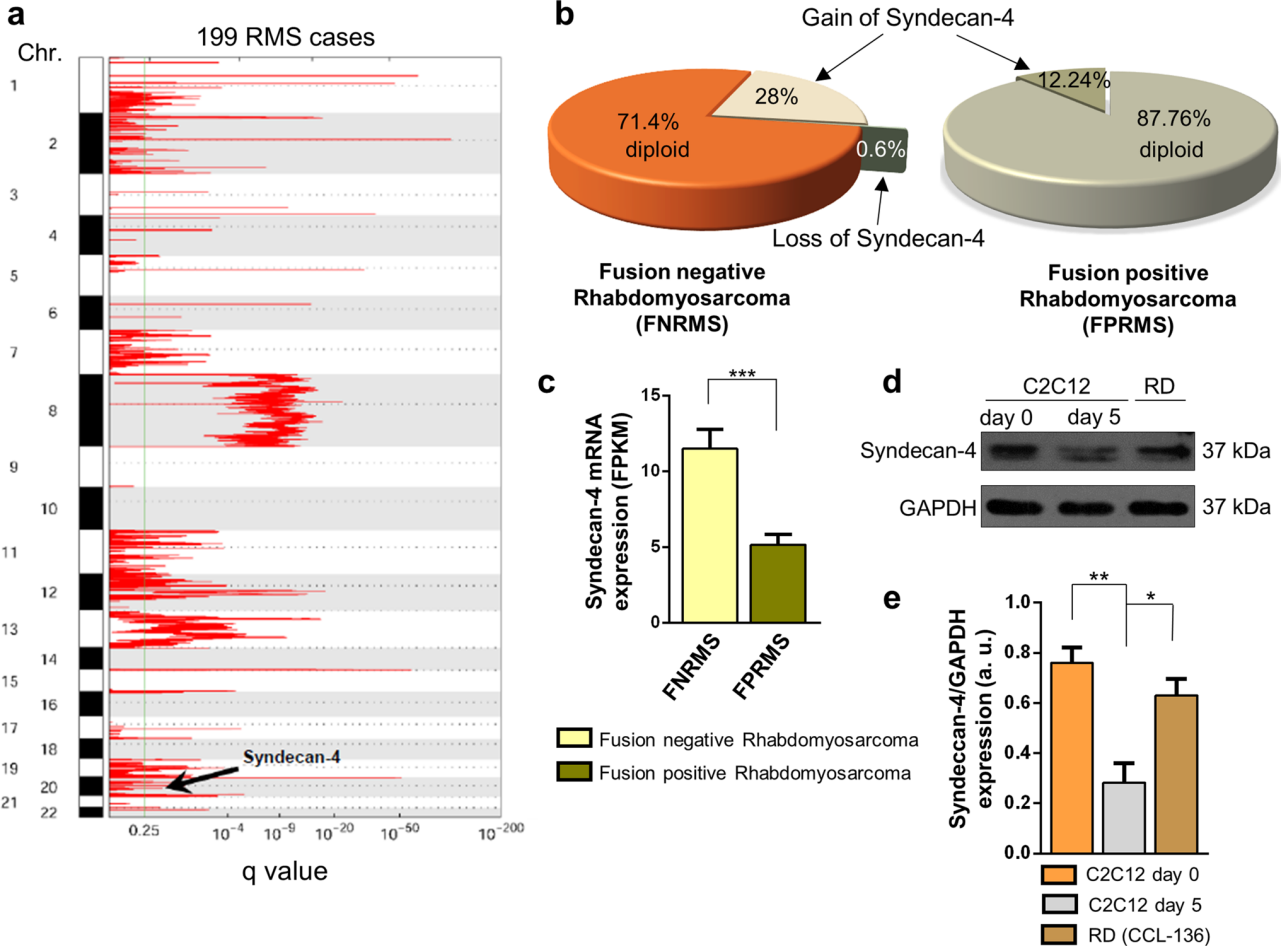
Syndecan-4 copy-number amplification and overexpression in human rhabdomyosarcomas. **a** A representative figure shows regions of the entire genome of rhabdomyosarcoma showing significant copy-number amplification, where the syndecan-4 site is designated (this is located on chromosome 20). *n* = 199 human rhabdomyosarcoma cases were analyzed. **b** Genomic analysis of fusion-negative rhabdomyosarcoma (FNRMS; *n* = 150) and fusion-positive rhabdomyosarcoma (FPRMS; *n* = 49) samples. Syndecan-4 copy-number amplification was observed in 28% of FNRMS cases that did not exhibit Pax gene fusion (**c**) RNA sequencing was performed, and syndecan-4 mRNA expression levels of FNRMS (*n* = 29) and FPRMS (*n* = 8) were quantified; mean + SEM; ***p < 0.001. **d** Representative immunoblot depicts the syndecan-4 expression of proliferating C2C12 myoblasts, differentiated C2C12 samples, and RD (fusion-negative rhabdomyosarcoma) cells. GAPDH was used as the loading control. **e** Quantification of western blot results is shown; *n* = 3 independent experiments; mean + SEM; **p < 0.01; *p < 0.05

Rhabdomyosarcoma is the most common form of pediatric soft tissue sarcoma, an aggressive tumor composed of myoblast-like cells. Based on our present study on the role of syndecan-4 in myoblast differentiation and considering the unknown role of syndecan-4 in rhabdomyosarcoma, we investigated the presence of syndecan-4 copy-number amplification and loss events in human rhabdomyosarcoma samples (Fig. 8). A representative GISTIC plot showed significant copy-number amplification regions in the entire genome based on 199 human rhabdomyosarcoma cases (Fig. 8a). The syndecan-4 locus is designated on chromosome 20, which is marked as a region of copy-number amplification (Fig. 8a). According to copy-number analysis, syndecan-4 was highly amplified in rhabdomyosarcoma, especially in FNRMSs, as genomic analyses revealed copy-number amplification events in 28% of fusion-negative tumors (Fig. 8b). Among 49 FPRMS patients, 6 showed gain of syndecan-4, but none showed loss of syndecan-4; however, among 150 FNRMS cases, 42 showed gain of syndecan-4, and 1 showed loss of syndecan-4. Based on the mRNA sequencing data, FNRMS cases were accompanied by increased syndecan-4 mRNA expression (Fig. 8c) compared to that in FPRMS cases, suggesting syndecan-4 as a potential tumor driver gene in FNRMS promoting tumorigenesis.

We compared syndecan-4 expression in C2C12 myoblast cells cultured in growth medium, differentiated C2C12 myotubes (cultured in differentiation medium for 5 days) and RD cells (Fig. 8d). Remarkably, RD cells are FNRMS cells. A representative immunoblot illustrated that syndecan-4 expression was reduced in differentiated C2C12 myotubes compared to that in proliferating C2C12 myoblasts. In addition, RD cells exhibit high syndecan-4 expression, which is almost comparable to that of proliferating C2C12 myoblasts. The observed high syndecan-4 expression in RD cells is consistent with the copy-number amplification and high mRNA expression of syndecan-4 in FNRMS tumors.

## Discussion

Skeletal muscle regeneration is a multistep process initiating from satellite cells and then leading to the formation of myotubes through myoblast fusion. Several conserved transcription factors and signaling molecules have been identified to regulate myogenesis, but their upstream regulators have been less characterized. In this study, we investigated the role of syndecan-4 in myoblast differentiation and fusion, because it is known that skeletal muscle regeneration is impaired in syndecan-4 deficient mice [36], although the exact mechanism has not been completely elucidated.

The first step in skeletal muscle regeneration is the proliferation of satellite cells. Our previous study showed that myoblast proliferation requires high syndecan-4 expression [23]. However, syndecan-4 is not only involved in skeletal muscle regeneration, but it is also involved in skin reepithelialization and vascular regeneration [53].

The essential role of syndecan-4 in muscle regeneration is supported by the experimental results published by Cornelison et al. [36]. They described that the absence of syndecan-4 reduces the degree of barium chloride-induced muscle regeneration compared to that in the wild type. Comparing normal and syndecan-4 KO mice, Ronning et al. revealed decreased MyoD and MyoG expression and smaller myotube cross-sectional area in syndecan-4 KO [36, 54]. Importantly, during in vivo studies, the migratory ability of the cells also have high impact for the fusion events. Our previous results indicated that silencing syndecan-4 expression reduces the migration of mammalian myoblasts in vitro [26, 27], which may explain the reduced regeneration and myotube formation in syndecan-4 KO mice [36, 54].

Because in vitro differentiation of C2C12 myoblasts is induced in a confluent cell culture, the cell-to-cell fusion can be investigated separately from prefusion migration events, and the migration deficiency of the cells did not disturb myotube formation. In our recent experiments, we observed increased myotube formation and increased size of myotubes due to the silencing of syndecan-4 expression. Consistent with our results, Ronning et al. earlier reported an increase in myotube number after the administration of siRNA that silenced syndecan-4 expression; however, the desmin level showed no increase in their samples [37].

Moreover, syndecan-4 KO increases Rac1 GTPase activity in fibroblasts [33], and consistent with these results, we showed in the present study that silencing the expression of syndecan-4 increased Rac1-GTP levels in myoblasts. Importantly, Rac1 was reported to play an essential role in the fusion of mammalian myoblasts [12] and in the rearrangement of the actin cytoskeleton through PAK1 [9], which fundamentally determines cellular elasticity [52].

Syndecan-4 connects the extracellular matrix to the cytoskeleton, thereby allowing the interaction of the cell and matrix components, growth factors, or cytokines [55]. Syndecans play an important role in the formation of cell–matrix adhesion complexes together with transmembrane integrins; however, signaling kinases, e.g., focal adhesion kinase (FAK) and PKCα, and structural proteins (e.g., paxillin, talin, and vinculin) also play a role in the formation of focal adhesions. Integrins, especially β1 integrins, regulate myoblast fusion and sarcomere structure assembly [56]. Moreover, an increase in FAK (Tyr397) phosphorylation has been described in myoblast fusion [57]. In the absence of FAK, impaired fusion was observed, but no inhibition of myogenic differentiation occurred, suggesting that FAK plays a unique role in cell fusion [58]. Fibronectin forms a bridge between syndecan-4 and α5β1 integrins [17]. In mouse fibroblasts, the presence of syndecan-4 was found to regulate FAK (Tyr397) phosphorylation. Decreased phosphorylation levels have been detected in fibronectin-associated syndecan-4 KO cells, which affect the development and number of focal adhesions [17, 59]. Alpha-actinin is also a component of focal adhesions that is directly linked to the variable region of syndecan-4 [31]; thereby affecting contractility and actin cytoskeletal rearrangement. Hence, the proteins that constitute the cytoplasmic side of focal adhesions provide structural stability on the one hand and connect different signaling pathways on the other hand.

Cornelison et al. described that MyoD expression is reduced in satellite cells, and MyoD exhibits 60–80% of cytoplasmic localization in the absence of syndecan-4, whereas only nuclear localization is observed in the wild type [36]. In our study, we monitored the changes in MyoD expression during the differentiation of syndecan-4 cell cultures and observed a significant increase compared to that the wild type, suggesting increased differentiation.

The rearrangement of the actin cytoskeleton plays a vital role in the cell-to-cell fusion process. Although the regulation of cell–cell fusion events is conservative, the structure of actin-based protrusions is different in *Drosophila* and mammalian cells. In mammalian cells, finger-like protrusions develop in the fusion area [15] unlike the single actin spike (actin focus) of *Drosophila* cells [11]. Randrianarison-Huetz et al. described that Srf regulates the actomyosin network in mammalian satellite cells, which may contribute to the maintenance of mechanical stress or stiffness, allowing productive invasion and fusion along with actin-based protrusions [15]. Srf exhibits a pleiotropic role, including activation of MyoD expression, proliferation, and differentiation in the C2C12 cell line [15].

The remodeling of actin cytoskeleton is primarily regulated by members of the Rho family of small GTPases. The role of Rho GTPases has already been investigated in myoblast fusion as well. The cytoplasmic domain of syndecan-4 regulates Rac1 activity [33, 34]. Rac1 levels are increased at the site of fusion, and constitutively active Rac1 induces myoblast fusion [12]. In contrast, RhoA antagonizes Rac1, and constitutively active RhoA reduces myoblast fusion [13]. In our syndecan-4 knockdown samples, the phosphorylation of PAK1 and cofilin was also increased as a result of enhanced Rac1 activity, as lower levels were obtained after Rac1 inhibition (NSC23766 treatment), and the values were similar to those of the untreated wild-type C2C12 cell line. All these results indicate an intensive remodeling of the actin cytoskeleton in syndecan-4 silenced cells.

We visualized the rearrangement of the actin cytoskeleton by dSTORM superresolution microscopy. In our previous research [27], we investigated the changes in the nanostructure of the lamellipodial actin network of migrating cells after wound scratching, where both the number and length of branches were decreased in the lamellipodia after syndecan-4 silencing. In the present study, we analyzed the cortical actin network in fusing cell cultures and observed robust, thicker cortical actin structure in syndecan-4 silenced samples. In the case of mononuclear but nonfusing cells adhering to the substrate, we observed that the number of actin branches was increased, but their length was decreased in syndecan-4 silenced cells compared to controls. Several studies described that SRF affects actin cytoskeleton [15, 60, 61]. Regulation of actin dynamics is required for serum induction of a subset of SRF target genes, including vinculin or cytoskeletal actin [60]. According to our results, the serum content of the cell culture media (20% FBS vs. 2% horse serum) affected the actin nanostructure of the C2C12 cells. The syndecan-4 silenced cells exhibited decreased number of branches in 20% FBS, while increased number of branches were observed in 2% horse serum.

The actin cytoskeleton is known to play an important role in determining cell elasticity [52, 62]. A previous study emphasized the importance of examining the elastic properties of cells. Examining cell elasticity may help, among other aspects, in myocardial tissue replacements, where skeletal muscle myocytes with appropriate elastic properties are selected for implantation into the myocardium. This achieves appropriate functional integration of donor cells into the recipient tissue [63]. To the best of our knowledge, no study discussed the changes in syndecan-4 expression and elasticity in myotubes. Therefore, whether any relationship exists between syndecan-4 expression and myoblast elasticity is not clear. Our results indicated that silencing syndecan-4 expression reduced the elasticity of cells, increased their hardness, and could result in a stronger actin structure, which may even play a role in the mechanical basis of the fusion.

Members belonging to the syndecan family regulate cell adhesion, proliferation, and differentiation. The role of syndecans in tumor formation and progression has been extensively investigated. Of these syndecans, syndecan-1 is the most investigated prognostic marker in several tumor types [64]. Elevated expression levels of syndecan-1 have been reported in breast cancer, pancreatic cancer, and squamous cell carcinoma of the lung, whereas increased levels of syndecan-2 have been observed in melanoma and colon cancer [65]. Changes in syndecan-4 expression levels can be observed in several tumor types, and it serves as a prognostic marker, such as in breast cancer, glioma, melanoma, liver cancer, and osteosarcoma [65–67]. However, the role and expression of syndecan-4 in rhabdomyosarcoma have not been yet examined. Our previous results demonstrated that the high syndecan-4 expression levels in proliferating myoblasts are gradually decreased during differentiation [23]. According to our present study results, FNRMS samples exhibit a higher proportion of syndecan-4 copy-number amplification, and their syndecan-4 mRNA expression is higher than that of FPRMS samples. In addition to these results, western blot analysis of FNRMS cells revealed high levels of syndecan-4 at the protein level.

The molecular basis of FNRMS cases is highly heterogeneous. Other molecules, e.g., transcription factors such as Twist1 and Twist2, have already been described to act as oncogenes in FNRMS [48]. Moreover, the transcription factor PROX1 has been shown to be highly expressed in rhabdomyosarcoma [68]. Several prognostic markers have been identified, such as CD44, AP2 β, P-cadherin, epidermal growth factor (EGFR), and fibrillin-2 [69]. CD44 is a proteoglycan whose expression levels are altered in various tumors as well as in childhood malignant neuroblastoma and in rhabdomyosarcoma. The changes in its levels correlate with prognosis, where low expression correlates with poor outcome; therefore, investigating CD44 levels may be useful in selecting patients for treatment [70]. Nevertheless, other proteoglycans are also involved in rhabdomyosarcoma, such as chondroitin sulfate proteoglycan 4 (CSPG4) and glypican-3 (GPC3). CSPG4 is a predictive marker for poor-onset tumors such as breast cancer and soft tissue sarcomas [71]. Expression of GPC3 has also been demonstrated in rhabdomyosarcoma, but not in adult soft tissue sarcomas [72].

Interestingly, syndecan-4 was descibed as a target for antiancer drugs in different cell lines. The humanized recombinant monoclonal antibody trastuzumab, an inhibitor of ErbB2 (HER2), reduced syndecan-4 expression [67]. Moreover, panitumumab, a human monoclonal antibody inhibiting epidermal growth factor receptor (EGFR), also decreases the expression of syndecan-4 [73].

**Fig. 9:**
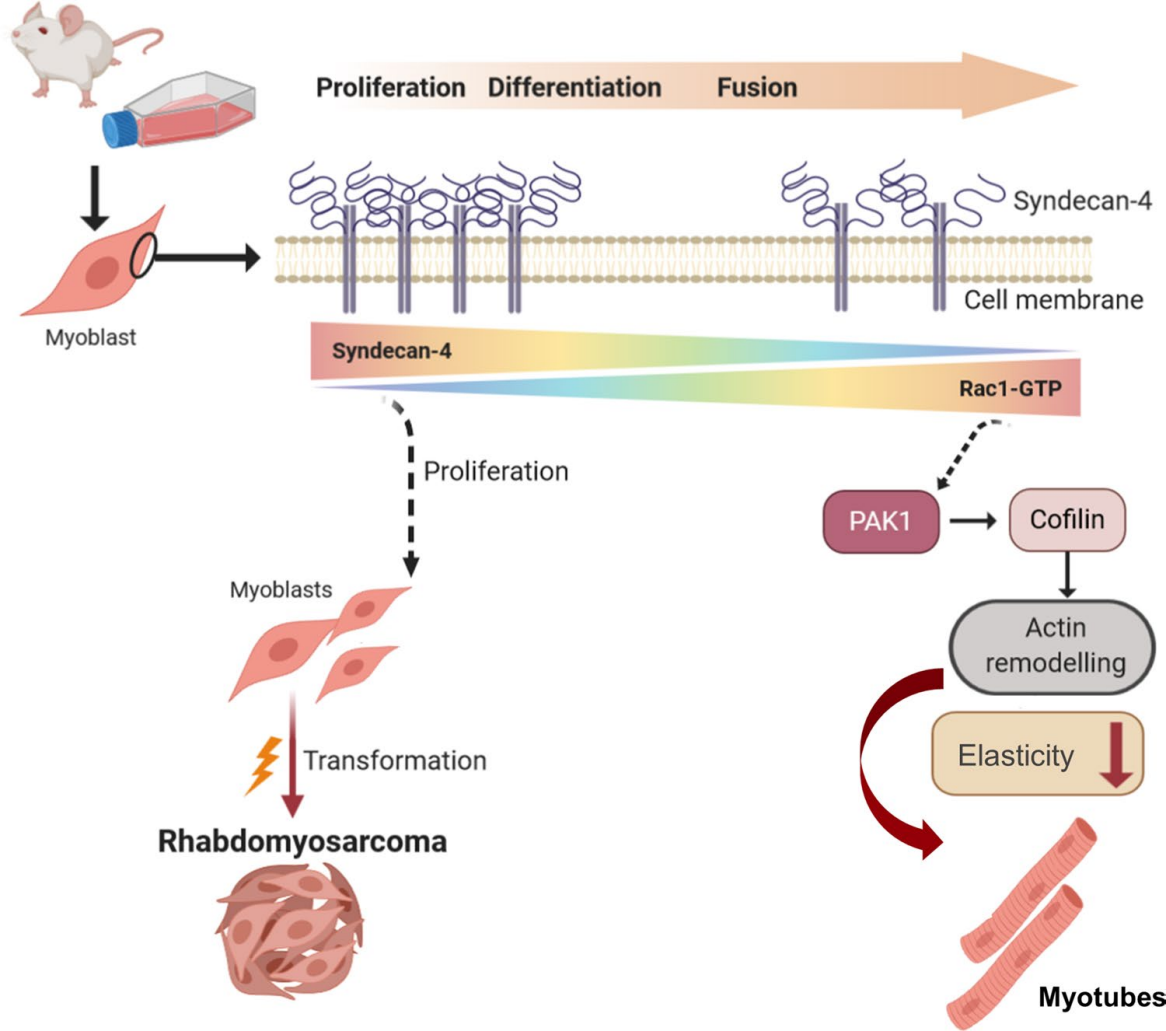
Schematic summary of the effects of syndecan-4 on muscle differentiation and tumorigenesis. Syndecan-4 expression gradually decreases during muscle differenctiation allowing Rac1 activation. As a consequence, the actin remodeling and the formation of a thicker cortical actin reduce cellular elasticity, thereby mediating myoblast fusion. High syndecan-4 expression inhibits myogenesis, and an increased syndecan-4 copy-number and mRNA level have been observed in tissue samples and rhabdomyosarcoma cells

To summarize, we described the role of syndecan-4 in muscle differentiation and its expression in rhabdomyosarcoma. The gradually decreasing levels of syndecan-4 during muscle differentiation allow Rac1 GTPase activation, and the syndecan-4/Rac1-mediated rearrangements of actin play a vital role in cell fusion. Thicker cortical actin was observed in syndecan-4 silenced myotubules, and the elasticity of these cells decreased; therefore, these cells were harder than the control cells. This may explain the increased fusion capacity of syndecan-4 silenced cells and hence their role in providing the mechanical basis for fusion. An increased syndecan-4 copy-number and mRNA level has been demonstrated in tissue samples and RD cells, but further in depth analysis required to elucidate the role of syndecan-4 in tumorgenesis (Fig. 9). Therefore, our results provide insight into the molecular etiology of rhabdomyosarcoma and syndecan-4 could be a potential drug target for this aggressive tumor group in the future.

### Supplementary Information

Below is the link to the electronic supplementary material.

Supplementary Fig. 1 Effect of syndecan-4 overexpression on C2C12 myoblasts. (a) C2C12 cells were stably transfected with syndecan-4 fused to GFP (SDC4::GFP) to increase the expression of syndecan-4. Representative phase-contrast images show the phenotype of cell lines. (b) Protein extracts of non-transfected C2C12 myoblasts and cells expressing SDC4::GFP were harvested at indicated time points of differentiation and subjected to SDS/PAGE. Representative immunoblots depict the expression levels of MyoD, MyoG, Cyclin E, Cyclin D, and p21. GAPDH was used as the loading control (TIF 6724 KB)

Supplementary Fig. 2 dSTORM analysis of the actin network of C2C12 myoblasts cultured in proliferation or differentiation media. (a) Representative dSTORM and skeletonized binary images of phalloidin-stained (Alexa 647, red) non-transfected C2C12 cell lines cultured in either proliferaion (20% FBS) or differentiation media (2% horse serum). (b) Schematic illustration of the actin strucure of the cells. (c) The number of branches, the normalized branch number, and the length of branches of the actin cytoskeleton were used to quantify changes in the actin nanosctructure (n = 4–12 independent experiments; mean + SEM; * p < 0.05; ** p < 0.01; **** p < 0.0001). Scale bar: 2 µm (TIF 14029 KB)

Supplementary file3 (DOCX 26 KB)

## Data Availability

The datasets generated and analyzed during the current study and all materials are available from the corresponding author on reasonable request. The original genomic data is deposited to dbGAP database with accession number phs000720.
