# Supplementary figures and images for: Syndecan-4 affects myogenesis via Rac1-mediated actin remodeling and exhibits copy-number amplification and increased expression in human rhabdomyosarcoma tumors
Source: Cell Mol Life Sci. 2022 Feb 7;79(2):122. doi: 10.1007/s00018-021-04121-0 (PMC8818642; doi:10.1007/s00018-021-04121-0)

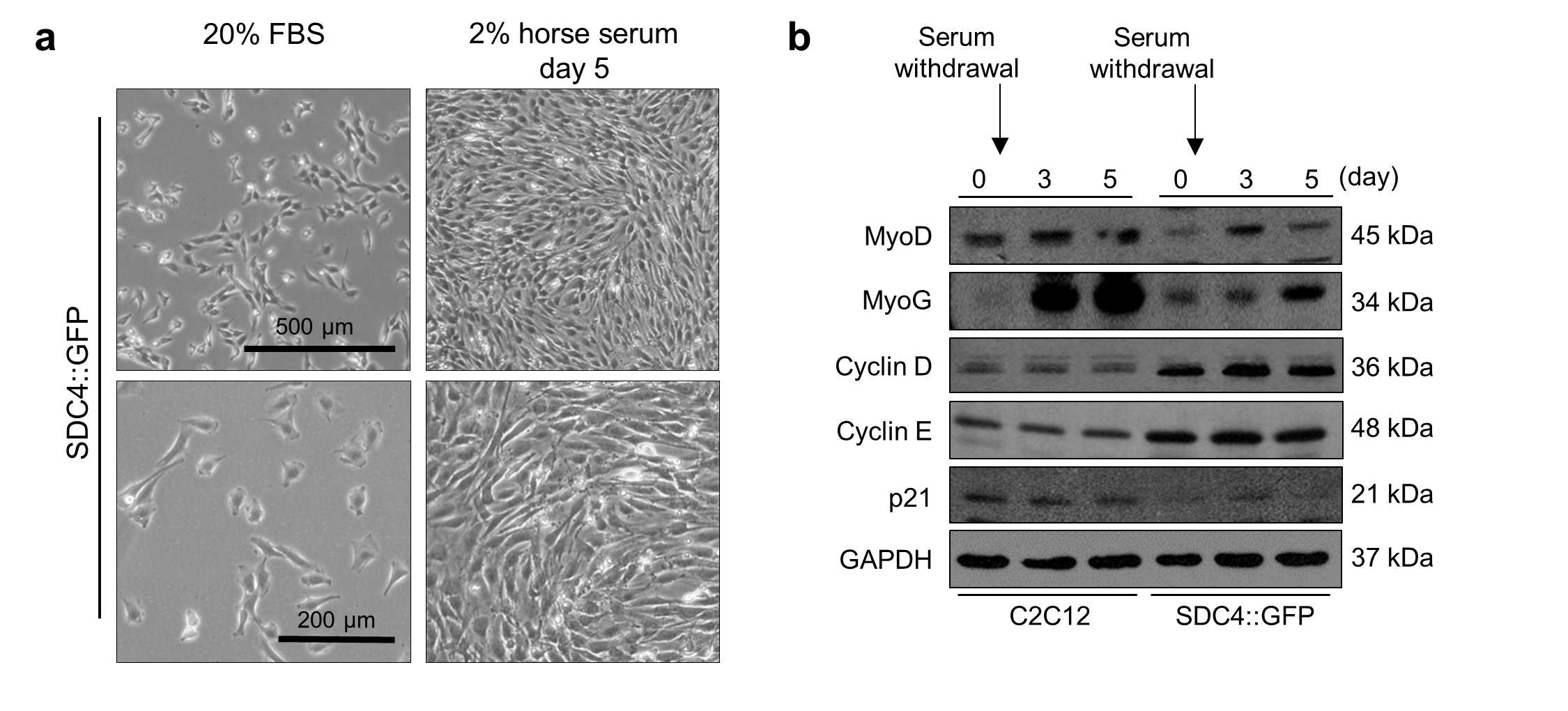

Supplement: Supplementary file 1 — Supplementary Fig. 1 Effect of syndecan-4 overexpression on C2C12 myoblasts. (a) C2C12 cells were stably transfected with syndecan-4 fused to GFP (SDC4::GFP) to increase the expression of syndecan-4. Representative phase-contrast images show the phenotype of cell lines. (b) Protein extracts of non-transfected C2C12 myoblasts and cells expressing SDC4::GFP were harvested at indicated time points of differentiation and subjected to SDS/PAGE. Representative immunoblots depict the expression levels of MyoD, MyoG, Cyclin E, Cyclin D, and p21. GAPDH was used as the loading control (TIF 6724 KB) [file 18_2021_4121_MOESM1_ESM.tif]

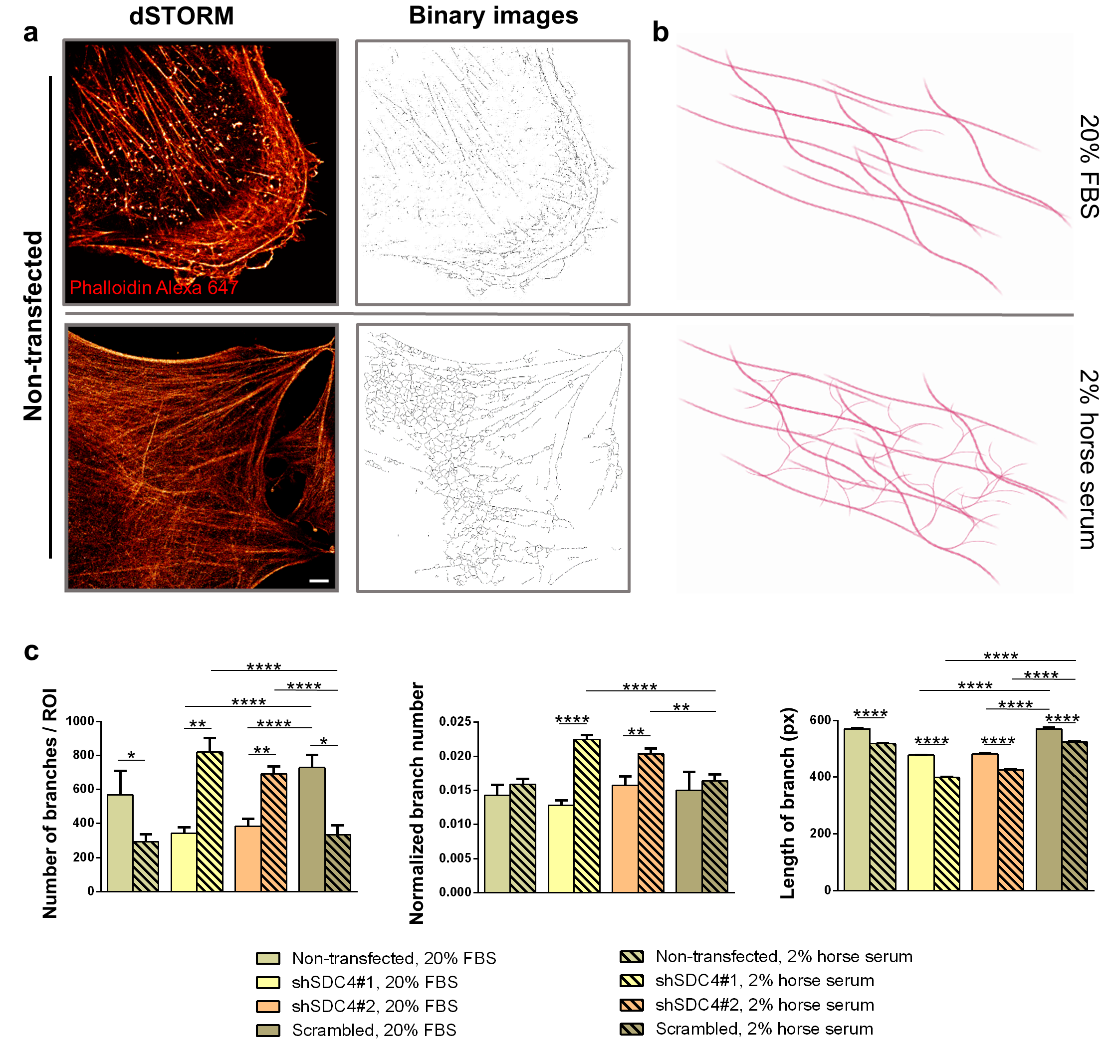

Supplement: Supplementary file 2 — Supplementary Fig. 2 dSTORM analysis of the actin network of C2C12 myoblasts cultured in proliferation or differentiation media. (a) Representative dSTORM and skeletonized binary images of phalloidin-stained (Alexa 647, red) non-transfected C2C12 cell lines cultured in either proliferaion (20% FBS) or differentiation media (2% horse serum). (b) Schematic illustration of the actin strucure of the cells. (c) The number of branches, the normalized branch number, and the length of branches of the actin cytoskeleton were used to quantify changes in the actin nanosctructure (n = 4–12 independent experiments; mean + SEM; * p < 0.05; ** p < 0.01; **** p < 0.0001). Scale bar: 2 µm (TIF 14029 KB) [file 18_2021_4121_MOESM2_ESM.tif]
