# Supplementary material for: Syndecan-4 affects myogenesis via Rac1-mediated actin remodeling and exhibits copy-number amplification and increased expression in human rhabdomyosarcoma tumors
Source: Cell Mol Life Sci. 2022 Feb 7;79(2):122. doi: 10.1007/s00018-021-04121-0 (PMC8818642; doi:10.1007/s00018-021-04121-0)
Supplement: Supplementary file 3 — Supplementary file3 (DOCX 26 KB) [file 18_2021_4121_MOESM3_ESM.docx]

**SUPPLEMENTARY METHODS**

**Cell culturing and transfection**

C2C12 mouse myoblasts (ATCC; Massanas, VA, USA) were stably transfected with plasmids expressing human full length syndecan-4 fused to green fluorescent protein (SDC4::GFP). Cells were transfected using X-tremeGENE (Roche; Basel, Switzerland) transfection reagent. Then. cells were selected in culture medium containing 80% DMEM (4.5 g/L glucose, l-glutamine, and pyruvate; Lonza), 20% FBS (Gibco), 50 µg/ml gentamicin, and 750 ug/ml G418 (Sigma-Aldrich) to generate stably overexpressing cell lines,. Stable cell lines were cultured in medium containing 500 ug/ml G418.
